# Medical device regulation and oversight in African countries: a scoping review of literature and development of a conceptual framework

**DOI:** 10.1136/bmjgh-2023-012308

**Published:** 2023-08-09

**Authors:** Naima Nasir, Sassy Molyneux, Fred Were, Adeniyi Aderoba, Sebastian S Fuller

**Affiliations:** 1 Health Systems Collaborative, Center for Global Health Research, Nuffield Department of Medicine, University of Oxford, Oxford, UK; 2 Health Systems and Research Ethics, KEMRI-Wellcome Trust Research Programme, Kilifi, Kenya; 3 Department of Paediatrics and Child Health, University of Nairobi, Nairobi, Kenya; 4 Kenya Paediatric Research Consortium, Nairobi, Kenya; 5 Reproductive, Maternal Health, and Healthy Ageing Unit, Universal Health Coverage-Life Course Cluster, World Health Organization Regional Office for Africa, Brazzaville, Democratic Republic of Congo; 6 Center for Global Health Research, Nuffield Department of Medicine, University of Oxford, Oxford, UK

**Keywords:** Health systems, Review

## Abstract

Regulatory and other governance arrangements influence the introduction of medical devices into health systems and are essential for ensuring their effective and safe use. Challenges with medical device safety, quality and use are documented globally, with evidence suggesting these are linked to poor governance. Yet, medical device regulation and oversight remain inadequately defined and described, particularly in low-income and middle-income settings. Through this review, we sought to examine the literature available on regulatory and oversight processes for medical devices in African countries.

Following a systematic approach, we searched academic databases including PubMed, Embase (Ovid) and MEDLINE (Ovid), supplemented by search for grey literature and relevant organisational websites, for documents describing medical device regulation and oversight in African countries. We summarised the data to present key actors, areas for regulation and oversight and challenges.

A total of 39 documents reporting regulation and oversight of medical devices were included for analysis. Regulatory and oversight guidelines and processes were reported as inadequate, including limited pre-market testing, reliance on international certifications and limited processes for post-market monitoring and reporting of adverse events. Challenges for regulation and oversight reported included inadequate funding, personnel and technical expertise to perform regulatory functions. The literature highlighted gaps in guidelines for donated medical devices and in information on governance processes at the national level.

The current literature provides a general overview of medical device regulatory guidelines and limited evidence on the implementation of regulatory/oversight processes at national and especially subnational levels. We recommend further research to elucidate existing governance arrangements for medical devices within African countries and propose a conceptual framework to inform future studies. The framework provides entry points for careful examination of governance and oversight in policy and practice, the exploration of governance realities across the health system and the influence of wider system dynamics.

**WHAT IS ALREADY KNOWN ON THIS TOPIC**

- Regulatory and other governance arrangements are recognised as critical for ensuring quality, safety and appropriate management of medical devices within health systems. However, medical device governance, including regulation and oversight is less defined and not well understood in low-income and middle-income countries, particularly in African countries.

**WHAT THIS STUDY ADDS**

- The review shows empirical evidence on medical device regulation and oversight in African countries is limited, with the current literature providing general descriptions of national-level guidelines and processes, and limited information on medical device governance and oversight at the subnational level.

**HOW THIS STUDY MIGHT AFFECT RESEARCH, PRACTICE OR POLICY**

- The study highlights the need for further research to understand governance arrangements for medical devices across all levels of the health system. We propose a conceptual framework to guide the careful examination of medical device governance and oversight and explore where current policy and practice might be improved.

## Introduction

Health technologies including medical devices have shown potential to improve the quality, safety and outcomes of healthcare delivery.^1 2^ However, the introduction and use of health technologies can be challenging, leading to poor adoption, improper use and unintended harms and risks.^2 3^ Governance arrangements, including regulatory and oversight structures and rules, can influence the selection, introduction, adoption and appropriate use of safe and good quality health technologies, which ultimately improves patient safety and health outcomes.^4^ Thus, regulatory and other governance arrangements are essential for ensuring the effective and safe use of health technologies in health settings, to maximise benefits and minimise risks and harms.^2 4^

Governance is well recognised as a critical function and building block of the health system.^5 6^ Although there is still no consensus definition, governance has been broadly defined as structures, processes and rules which are interpreted and applied across the various levels of the health system.^4 6 7^ Governance arrangements influence the functions and responsibilities of health system actors (including authority and decision-making, accountability, regulation and oversight) and the relationships between them.^4 8^ Recent approaches to examining governance in health systems move beyond focusing only on the formal structures of government, to also consider informal rules and processes.^7 8^ In addition, such approaches recognise the role of decentralisation in different settings, which shifts the roles and power of multiple actors (organisations, groups, and individuals) involved in the practice of governance across the health system.^7 9^

Governance arrangements for health technologies are influenced by how the technology is defined or categorised.^10 11^ Challenges with definitions and classification have been shown to influence regulation and oversight of *medical devices* specifically.^1 11^ The WHO defines a medical device as ‘any instrument, apparatus, implement, machine, appliance, implant, reagent for in vitro use, software, material or other similar or related article, intended by the manufacturer to be used, alone or in combination for a medical purpose’.^12^ Such a broad and complex definition results in a wide range of products, from—weighing scales to complex medical equipment,—being designated as medical devices. This has led to difficulties in classification and standardisation, varied regulatory regimes across countries and in some cases less rigorous regulatory approval processes for medical devices when compared with other health technologies such as medicines and vaccines.^11 13^

Concerns about the regulation and oversight of medical devices continue to be raised globally, owing to the significant number of medical devices found to be of poor quality, unsafe and abandoned across health settings.^14–17^ In African countries, medical devices are often donated or procured for use from high-income settings, are seldom designed for use in lower-resource settings and may not be appropriate for adoption and use.^15 16 18^ Other challenges such as mismatches between donations and recipient needs, incompatibility of devices with tropical environmental conditions, limited availability of enabling infrastructure such as space, power and water supply, inadequately trained personnel for proper use and maintenance and limited access to spare parts and accessories for long-term maintenance have been highlighted.^15 18 19^ There are also concerns about significant harm or risks to patients arising from the introduction of poor quality, obsolete or malfunctioning devices into health facilities and improper use by healthcare providers.^2 19–21^

Most of these challenges are linked to inadequate governance of medical devices, including regulation, oversight and management.^1 22 23^ Yet, in many low and low-middle income countries, including countries in Africa, the regulation of medical devices remains generally less well established and defined than those for other health technologies such as medicines and vaccines.^1 11 23–25^ In general, when compared with other regions, the WHO Regional Office for Africa (AFRO) region has more countries without a regulatory framework or a national regulatory authority (NRA) responsible for medical devices.^25 26^ Where frameworks and NRAs exist, reports highlight challenges that hinder regulatory systems and processes from functioning adequately.^23 26^ Furthermore, it is unclear how medical devices approved by NRAs for introduction into markets are adopted in health facilities, and what governance and oversight processes may be applicable beyond national-level processes.

Overall, medical device regulation and oversight processes within Africa are not clearly understood. This scoping review explored the nature and extent of the literature available on how medical devices are regulated and overseen, as part of governance arrangements within health systems in African countries.

## Methods

The scoping review was conducted following the guidance provided by the Joanna Briggs Institute Manual for Evidence Synthesis,^27^ which builds on earlier guidance on scoping reviews provided by Arksey and O’Malley,^28^ Levac and colleagues^29^ and Peters *et al*.^30^ In addition, guidance on the conduct and reporting of scoping reviews as outlined in the Preferred Reporting Items for Systematic review and Meta-Analysis extension for Scoping Reviews (PRISMA-ScR) guidelines (online supplemental appendix S3) was followed.^31^ Following an initial exploratory search of the literature, a protocol (online supplemental appendix S5) was developed with the following questions to guide the review:

10.1136/bmjgh-2023-012308.supp3 Supplementary data

10.1136/bmjgh-2023-012308.supp5 Supplementary data

1. How are medical devices categorised and defined in the literature for regulation and oversight?
2. What regulatory guidelines and processes are in place in African countries, and do these differ by categorisation/classification of medical devices?
3. How are governance processes implemented at the national and subnational levels, and which are the actors involved?
4. What are the challenges and opportunities for improving regulatory and oversight guidelines and processes?

### Search strategy and sources of literature

To identify keywords related to the review topic and refine the search strategy, a preliminary database search was conducted in MEDLINE and PubMed in July 2021. Following this, a definitive search to identify relevant publications was conducted between July and August 2021 in seven databases including PubMed, Embase (Ovid), Scopus, MEDLINE (Ovid), Web of Science, CINAHL (EBSCOhost). Keywords and search terms for the review included ‘medical devices’, ‘regulation’, ‘governance’, ‘oversight’, ‘sub-Saharan Africa’ and their synonyms. The search terms, including keywords and MeSH (Medical Subject Headings) terms for PubMed, are shown as an example in box 1. The final search strategies are provided in online supplemental appendix S4.

10.1136/bmjgh-2023-012308.supp4 Supplementary data

Box 1 **Search terms and key words for PubMed:**

((((“Equipment and Supplies”(MeSH))) OR (“medical device*“(Title/Abstract))) AND ((“Africa South of the Sahara”(MeSH)) OR ((“Subsaharan Africa”(Text Word) or “Sub-Saharan Africa”(Text Word) or Angola or Benin or Botswana or “Burkina Faso”(Text Word) or Burundi or “Cabo Verdi”(Text Word) or Cameroon or “Cape Verde”(Text Word) or “Central African Republic”(Text Word) or Chad or Comoros or Congo or “Cote d'Ivoire”(Text Word) or Djibouti or “Equatorial Guinea”(Text Word) or Eritrea or Eswatini or Ethiopia or Gabon or Gambia or Ghana or Guinea or Guinea-Bissau or Kenya or Lesotho or Liberia or Madagascar or Malawi or Mali or Mauritania or Mauritius or Mozambique or Namibia or Niger or Nigeria or Reunion or Rwanda or “Sao Tome”(Text Word) or Principe or Senegal or Seychelles or “Sierra Leone”(Text Word) or Somalia or “South Africa”(Text Word) or Sudan or Swaziland or Tanzania or Togo or Uganda or “Western Sahara”(Text Word) or Zambia or Zimbabwe) OR (“east africa*“(Text Word) OR “central africa*“(Text Word) OR “southern africa*“(Text Word))))) AND ((regulation(Title/Abstract) OR oversight(Title/Abstract) OR governance(Title/Abstract) OR “regulatory framework*“(Title/Abstract) OR “regulatory establishment”(Title/Abstract) OR “regulatory process*“(Title/Abstract) OR “regulatory authorit*“(Title/Abstract) OR “regulatory capacity”(Title/Abstract)) OR ((“Device Approval”(MeSH)) OR “Government Regulation”(MeSH)))

MeSH, Medical Subject Headings.

Similar keywords and terms were used to search other non-indexed databases such as Science Direct, African Journals Online, African Digital Archive and Policy Commons. Furthermore, a grey literature search was conducted using Google and Google Scholar, including a search through the WHO website, identified as a key organisation involved in medical device regulation in Africa. The reference lists of included studies were searched to identify additional material of relevance for inclusion in the review.

### Eligibility criteria

#### Eligibility criteria and selection of sources of evidence

The criteria for inclusion and exclusion of the sources of evidence are shown in table 1 below. The results from the searches were collated and screened in an iterative process by two reviewers. First, a title and abstract screen were performed, followed by an assessment of full-text articles to further determine eligibility for inclusion in the review. Any disagreements regarding eligibility were resolved by discussion between both reviewers, reaching a consensus. Where consensus could not be reached, a third reviewer resolved disagreements. Covidence, a web-based collaboration software platform that streamlines the production of systematic and other literature reviews^32^ was used to manage the screening process, supplemented by the Microsoft Excel application.

**Table 1:** Eligibility criteria for selection and inclusion of studies in the review

Type of documents for inclusion	Scientific peer-reviewed articles, frameworks and guidelines, dissertation/thesis, any commentaries on regulatory policies and empirical papers reporting experience implementing regulatory policies/guidelines.
Exclusion criteria	Literature detailing regulation and oversight of medicinal products, medicines or pharmaceuticals, vaccines and any health technology that is not a medical device was excluded unless there is overlapping relevant information. Articles reporting clinical trial or regulatory approval data on efficacy or safety profiles of specific medical devices in human or animal populations. Literature only providing details of specific country legislation for medical device regulation.
Period	No time limits set.
Language	Only studies published in English were included.
Context	The broad context of the papers included in this review was the African context. Literature on the regulation of medical devices outside the African context was included where it provided information relevant to medical device regulation and oversight in Africa, such as the WHO and other global guidelines.
Concept	The concept of this review is the governance of medical devices—including the regulation and oversight of medical devices. The review included resources which either focus on or include information on regulation and oversight rules and processes.

### Data charting and synthesis

To extract study characteristics and other relevant details, a data collection tool was developed. Data extracted included study characteristics such as authors, publication year, publication type and country/region of focus. Also extracted were information on, available regulatory frameworks, medical device definition, device classification, core regulatory functions/phases, actors involved, challenges with, and opportunities to improve regulation and oversight. These categories were based on themes within included studies and a previous WHO report which identified key areas for the assessment and regulation of medical devices.^33^ The first reviewer extracted the data and a proportion (about 30%) was independently checked by the second reviewer for consistency.

As the main purpose of this review was to broadly map the existing literature, a formal assessment of the methodological quality of the included studies was not undertaken. Data extracted were summarised using figures, tables and narrative summaries.

### Stakeholder consultation

As part of the scoping review methodology, consultations with three researchers identified through our networks for their experience within the field of health system governance and implementation of health technologies in African countries were held. Discussions followed the initial search and study selection process, to help refine the search strategy and identify any key missing resources. These researchers were also consulted to share the findings and provided useful insights for the synthesis of findings.

## Results

### Summary of the literature search and included studies

The systematic search and screening of literature resulted in a total of 444 publications identified. Of these, 39 were included in the review for analysis. These include 10 journal articles,^34–43^ 3 guidelines,^44–46^ 21 reports,^47–52^ 2 conference reports/proceedings,^53 54^ 1 book chapter,^55^ 1 dissertation^56^ and 1 web page.^57^ The year of publication ranged from 2003 to 2021.

**Figure 1:**
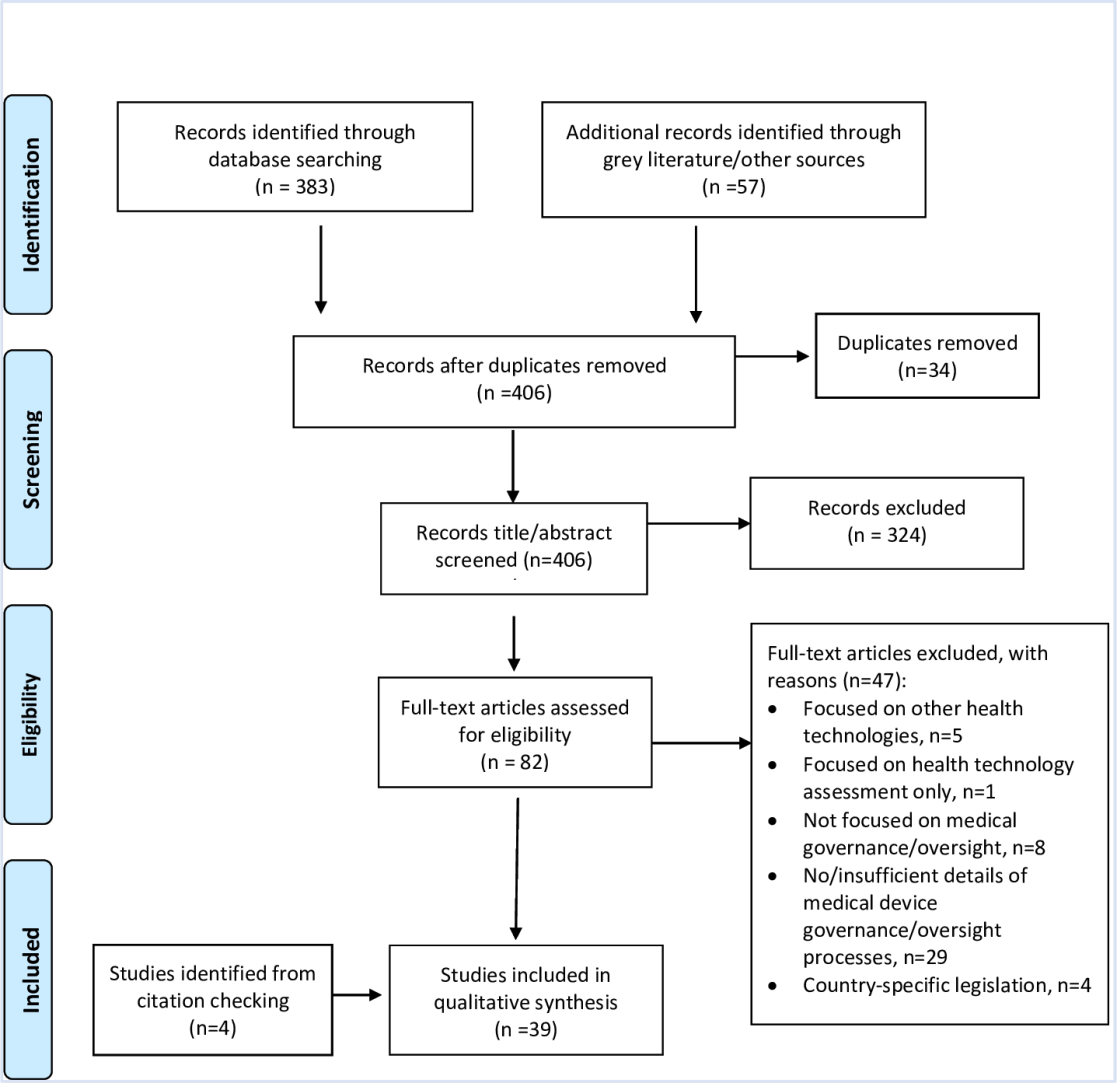
Preferred Reporting Items for Systematic review and Meta-Analysis extension for Scoping Reviews chart of selection and screening process.

Figure 1 below summarises the selection and screening process using the PRISMA-Scr.^31^ Online supplemental appendix S1 provides the characteristics of selected literature and online supplemental appendix S2 shows mapping of medical device governance, including regulation and oversight from the included literature.

10.1136/bmjgh-2023-012308.supp1 Supplementary data

10.1136/bmjgh-2023-012308.supp2 Supplementary data

### Medical device definitions

The most common definition was the WHO-Global Harmonization Task Force (WHO/GHTF) harmonised definition,^45^ (see box 2), which was cited by 13 publications.^37 40 42–44 46–48 55 56^ In some definitions, a separate definition for in vitro (outside the body) diagnostics (IVDs) was provided for medical devices used for the collection and examination of specimens taken from the body.^35 39 45^

Box 2 **WHO-Global Harmonization Task Force definition of medical device^45^**

Medical device means any instrument, apparatus, implement, machine, appliance, implant, in vitro reagent or calibrator, software, material or other similar or related article, intended by the manufacturer to be used, alone or in combination, for human beings for one or more of the specific purposes of:

- Diagnosis, prevention, monitoring, treatment or alleviation of disease.
- Diagnosis, monitoring, treatment, alleviation of or compensation for an injury.
- Investigation, replacement, modification or support of the anatomy or of a physiological process.
- Supporting or sustaining life.
- Control of conception.
- Disinfection of medical devices.
- Providing information for medical purposes by means of in vitro examination of specimens derived from the human body, and which does not achieve its primary intended action in or on the human body by pharmacological, immunological or metabolic means, but which may be assisted in its function by such means.

### Classification of medical devices

The key criteria used for the classification of medical devices was the risk of the medical device, that is, the potential of a medical device to cause harm or injury to the patient/end-user while in use.^35 38 45 46^ The end user was not clearly defined, but patients in healthcare settings such as clinics and hospitals, healthcare providers and device users outside the healthcare setting such as in homes were reported.^34 38 45^ The group or class of medical devices determined the degree of regulatory control and oversight required to ensure its safety, quality and performance.^45^ In general, the degree of regulatory scrutiny increased as the potential risks associated with the medical device increased, with low-risk devices subject to less scrutiny than devices considered to be of medium to high risk.^34 39 40 46^

From the literature, most countries in Africa used a four-group/tier classification system.^34 37 55^ These countries include Nigeria, Kenya, South Africa, Tanzania, Egypt, Sudan, Ghana, Morocco, Ethiopia and Uganda.^34 37 47 49 51^ These classification systems ranked devices from low risk to medium to high risk (designated as A, B, C and D or I, IIa, IIb and III), which aligned to either the GHTF classification which uses the A–D system or the US Food and Drug Administration (US FDA), which uses the I–III system.

### Actors involved in the regulation and oversight of medical devices

Several actors were described in the literature. Nearly two-thirds (n=31) of the included studies reported an NRA.^34–37 43 44 46–51 53 55 56^ Other actors reported include international organisation/agencies (WHO, United Nations, US FDA, European Medicines Agency, African Society of Laboratory Medicine, African Biomedical Engineers Consortium),^34 35 40 42 45 52 53^ national agencies such as Ministries of Health, Bureau of Standards, National Reference Laboratories, national disease control programmes,^35 42 43^ medical product procurement agencies, central medical stores within countries involved in medical device supply chain,^35 44^ manufacturers and vendors of medical devices,^38 40 44 45^ regulatory harmonisation working groups (Pan African Harmonisation Working Party, International Medical Devices Regulators Forum)^34 39 43 57^ and healthcare providers.^35 40 43 44^

The roles of these actors were not adequately described in the literature. For NRAs, the availability of a legal framework was cited as important, as this provided scope and legal backing for the NRA’s activities and empowered it to enforce its regulations.^45 46^

### Regulation and oversight of medical devices

#### Pre-market regulatory controls

Pre-market controls were concerned with the assessment of safety and functionality/performance of the medical device, and quality systems for the manufacture of the product.^35 39 40 45 46 56^ For diagnostic devices, the precision and accuracy (including sensitivity, specificity, and reproducibility of results) were also considered.^35 39^

These device attributes were usually assessed before gaining approval to market the device in a country or region, and manufacturers took primary responsibility to ensure conformity with the regulatory requirements and standards.^40 45 56^ National regulatory guidelines and policies set out the standards required, including testing methods and criteria for acceptance of conformity, with countries relying on standards set by International Standards Organisation (ISO13485:1996 and ISO13488:1996).^34 45^ To demonstrate conformity with regulatory requirements and standards, manufacturers were required to submit technical documents to the NRAs.^45–47 49^ Where required, the WHO guideline also recommended audits and physical inspections by regulatory staff to confirm what is submitted in the technical documentation for registration.^46^

For most countries, pre-market regulatory assessments were reported as limited or absent.^37 43 55^ Few countries were reported in the literature to have a pre-market regulatory assessment as part of the medical device regulatory systems. These include: Kenya,^43 51 56^ Tanzania,^43 47 55 56^ Ghana,^49 55^ Togo,^48^ Rwanda,^48^ Burkina Faso,^47^ Ethiopia,^47^ Morocco,^47^ Zimbabwe,^50^ Nigeria,^47^ Egypt^47^ and South Africa.^37 47 55^ Pre-market regulations varied from country to country, thus manufacturers seeking to register a product in several African countries would need to consider the unique regulatory requirements for market introduction in each country.^34 38^

Further details of the pre-market assessment of medical devices were limited. Requirements for clinical trials of medical devices were reported in a few countries including Zambia, Burkina Faso, South Africa, Tanzania^47^ and Ghana.^49^ These included obtaining ethical clearance and approval, either as part of the device development process or when introduced as part of research. Regulatory assessments generally did not consider clinical efficacy or cost effectiveness.^39^

Overall, the literature suggests most medical devices enter African country markets with limited pre-market regulation and may be sold and used without further testing of performance or safety.^35 40 52^ Medical devices used in disease-specific programmes, such as HIV, tuberculosis and malaria, which include test kits, condoms and/or gloves had more clearly defined regulatory processes, including testing of such products at national/HIV reference laboratories and established guidelines for registration and procurement.^35 37 43^ This appeared to be related to the involvement of international organisations providing such medical devices as part of their programmes but reasons for this were unclear.^42 43 46^

#### Reasons for limited pre-market assessment and regulation

Among the included literature, the most common reasons reported related to inadequate pre-market assessments were: limited scientific/technical capacity to conduct the necessary assessments and the high costs of regulatory evaluations,^38 41 45 46^ challenges with obtaining necessary licenses to apply international standards due to high costs^34 36 53^ and difficulties with conducting audits and inspections of manufacturing facilities for medical devices located outside the country or region.^34 37 55^ Only two countries, Ghana and Tanzania, were reported to have conducted audit inspections of manufacturing facilities outside their jurisdictions as part of pre-market assessment.^55 56^ One study also reported regulatory personnel had limited training for testing and evaluation of medical devices and as such undertook mainly desk reviews of product dossiers, checks of ISO and other certifications and risk-based classification of devices.^56^

Two studies reported tensions existing between agencies/organisations responsible for the regulation of medical products, resulting in a lack of clarity of regulatory requirements and duplication of responsibilities.^37 43^ The literature also suggests not all NRAs have adequately extended the scope of regulation and activities beyond pharmaceuticals/medical products/food/cosmetics to include regulation of medical devices.^34 36 55^

#### Reliance and recognition of international certification and standards

Studies reported African countries relied on or recognised the certification or evidence of pre-market assessments conducted by the NRAs and/or third-party organisations (referred to as notified bodies) of other countries such as the USA, Canada, Japan, Australia and the European Union (EU).^35 36 43 45 55 56^ Routine acceptance of the regulatory evaluations of internationally recognised institutions was reported as a means to provide simpler and more reliable pre-market regulatory approval of imported medical devices.^35 43 45^ However, one study described such international regulatory processes as insufficient for the regulatory oversight of medical devices, citing a lack of transparency, partiality towards manufacturers and limited evidence for the assessment of high-risk devices.^56^ The WHO prequalification assessment was also recognised across several African countries as a mechanism to ensure the quality of IVDs procured from manufacturers and as a requirement for donor agencies supplying medical devices.^35 39 42 56^

#### Placing-on-market regulatory controls

Placing-on-market regulations were closely related to the pre-market phase, concerned with the distribution and sale of medical devices in countries and included import controls, vendor establishment or registration, the listing of products, advertising controls (including appropriate labelling) and any after-sale vendor obligations.^45 46^ Studies reported regulations and controls for placing medical devices on the market were available in several African countries.^37–40 43 47–51 55^ Manufacturers and vendors of medical devices were required to register a local agent or representative within the country,^37 42 43 47^ list the products to be sold or distributed,^47^ obtain certification or approval for import, sale and distribution^37 47 48 50 51 55^ and ensure correct and appropriate information about the device’s performance, safety and intended use was provided to end users.^39 40 47^

As with pre-market regulation, several countries recognised or accepted certificates issued by institutions such as the US FDA, WHO prequalification and the EU *CE* mark/certificate of free sale, often without any additional in-country regulatory processes.^34 35 39 43 56^ The use of internationally recognised certifications helped to reduce costs and accelerate the regulatory process.^39 53^ International certifications could also be accepted following some verification of conformity to standards before the importation and shipment of medical devices.^43 53 55^ Verification processes were not clearly defined but one report highlighted the use of inspection visits to warehouses and facilities manufacturing medical devices and regulators in Tanzania and Uganda were reported to conduct such inspections.^43^

#### Post-marketing regulatory controls

The terms post-marketing surveillance (PMS), marketing surveillance and vigilance were used interchangeably in the literature to refer to post-market regulations and controls. Overall, PMS or vigilance activities referred to ongoing monitoring of the quality, safety and performance of medical devices while the product is in the market or used.^39 40 42 44 46^

Some countries were reported to have guidelines for PMS which generally described the responsibilities of the manufacturer, vendor or authorised representative for reporting adverse events, control of advertising and labelling and taking field safety corrective actions.^37 47–51^ Reports showed manufacturers and vendors were generally required to monitor the performance and safety of medical devices after sale or distribution, and feedback from manufacturers/vendors and users to the national regulatory agency was a key part of the post-market vigilance system.^40 44 45^ However, activities related to PMS and market vigilance in African countries were reported to be inadequate, including tracking of medical device safety and performance after the sale and while in use.^37 43 56^

Most countries were reported to have limited capacity for post-market regulation, including the inability to conduct quality tests on batches of products in the field and inadequate mechanisms for recall or withdrawal of substandard medical devices.^37 39 40 43 56^ Few countries were reported to conduct routine inspections of manufacturing facilities and warehouses to monitor the quality of devices produced (Morocco,^47^ Kenya,^51^ Egypt,^47^ South Africa^37 47^ and Tanzania^47^). A reactive approach to post-market regulation was described, which involved ad-hoc reporting of adverse events.^43 55^ Availability of systems for collecting feedback and information was limited; only Tanzania and Nigeria were reported to have mechanisms for collecting feedback on adverse events of medical technologies, including medical devices and communicating recall of substandard products.^43 47^

### Regulations for donated devices

Although problems with donated devices were highlighted in the literature, there was limited information in the included studies on specific guidelines or policies for oversight of donated devices within countries in Africa. Donated devices were generally described as subject to similar controls and requirements for safety, quality and performance as other devices imported through the regular supply chain.^37 46^ One study mentioned the presence of guidelines or policies for donated devices in eight countries in Africa but did not give further details.^34^ The same study also recognised the absence of regulations or policies for donated devices as a challenge.^34^

Few studies reported guidelines set by the WHO for donations of healthcare equipment to low-income settings, and regulatory support provided by international donors/organisations for medical devices donated through national disease programmes such as tuberculosis, HIV, and malaria.^38 43 45^

### Challenges of regulation and oversight of medical devices

Across the three phases of regulation and oversight, some cross-cutting challenges to regulation and oversight of medical devices were highlighted in the literature.

First, several studies highlighted medical device regulation was inadequate and challenged by limited funding, inadequate personnel and limited technical expertise and capacity of national regulatory authorities for oversight.^34 37–40 52 55^ Factors such as limited funding to apply international standards and guidelines,^53^ and limited training and capacity building of regulatory personnel to conduct rigorous assessments of medical devices were reported.^34 35 38 40 43^

Next, clear and specific guidance for regulatory oversight of donated and refurbished medical devices and equipment was lacking despite the influx of medical devices and equipment into African countries.^41 54^ Such devices were often found to be in poor working condition or abandoned.^54^ Concerns were raised regarding the quality of such devices, their appropriateness for low resource settings (given differences in design standards and technical specifications) and resilience to the more tropical environments found in African countries.^41 45 54^ Mismatch with local infrastructure, limited testing and inadequate resources/expertise to maintain equipment locally were cited as challenges.^37 54^

Another challenge was the regulatory approach for medical devices in African countries largely focused on pre-market assessment (including placing-on-market controls) at the national level and less on post-market oversight. This was raised in recognition of problems with medical devices arising while in use where oversight and vigilance were reported as inadequate.^36 56^

Finally, the diverse and varied regulatory requirements across African countries were thought to contribute to the limited access to medical devices in the region. Multiple regulations increased the costs and complexity of the registration process for manufacturers and vendors seeking to introduce devices into African markets.^37–40 52 55 56^ Information about regulatory requirements was described as limited and lacking transparency, with such variations resulting in lengthy approval processes and unnecessary duplication.^39 55 56^ This also served as a disincentive for local medical device development and innovation.^38 55^

### Opportunities to improve regulation and oversight of medical devices

Harmonisation of regulatory processes and requirements was the main opportunity for improving regulation and oversight of medical devices reported in the included publications.^34–40 42 43 45 52 54 56^ The adoption of a unified set of registration requirements and format for the submission of dossiers for pre-market approval was described as means to reduce regulatory barriers and increase access to medical devices.^37 39 40 55^ Harmonised guidance was also thought to provide clarity to medical device manufacturers about the registration processes, improve efficiency, reduce unnecessary duplication and costs and shorten the time for approvals.^39 40 43^ Similar regional harmonisation efforts have been successful in other regions such as Asia.^58^

Within African countries, the East African Community and the South African Development Community were identified as working towards harmonisation of regulatory guidelines, but this was largely focused on medicines and less on medical devices and diagnostics.^36 39 43 52 56^ The Pan African Harmonization Working Party, was reported as the main group coordinating regulatory harmonisation activities for health technologies including medical devices in Africa.^39 52 56 57^ However, no reports of existing harmonised regulations for medical devices in Africa were found in the literature.

## Discussion

Despite the recognition of the critical role of governance of medical devices in improving the quality of healthcare delivery, this review found limited published empirical literature on governance, including regulation and oversight, of medical devices in African countries. Although some relevant sources of evidence outside published empirical literature may have been missed, the findings highlight that several countries in Africa have country specific guidelines and frameworks for the regulation and oversight of medical devices, with at least 18 countries having a national regulatory framework.^34 37 43 47 48 53^

### Summary of findings

Along with the reliance on and recognition of certifications from higher-income settings, most of the existing regulatory guidelines and processes in African countries were modelled after guidelines from such settings (including the EU and US FDA). The findings suggest medical devices with international certifications are viewed as ‘trusted sources’ of quality, performance and safety thus not requiring further scrutiny. However, previous literature highlights regulatory processes governing medical devices in high-income countries also have shortcomings.^17 20 21^ We also found medical devices introduced by organisations implementing global health programmes benefitted from clearly defined processes for registration (using international certification) and support for further testing and evaluation.^35 37 43^ Although recognition of international certification can contribute towards regulatory efficiency, there is a clear need to focus efforts towards building local infrastructure and capacity to conduct regulatory evaluations within African countries.

While high-income settings may be able to implement pre-market testing and approval requirements set in those contexts, the review findings indicate much of this regulatory guidance remains poorly implemented and ineffective in lower-resource settings. This may be due to challenges highlighted in this review and previous literature, including funding constraints, inadequate personnel and a lack of technical expertise among other factors.^15 18 26^ Furthermore, the regulatory needs of countries in Africa, where most medical devices are imported or donated will likely differ from the needs of higher-income countries which have substantial capacity and expertise for the manufacture and export of medical devices.^23^ As such, the applicability of such regulatory guidance in African contexts has been questioned.^1 23 33^ This review brings to fore such questions, and the need for local regulatory regimes to reflect the contextual realities and practicalities of medical device governance in African settings.

Overall, the findings indicate the core regulatory functions for medical devices identified were inadequate, ineffective and weakly enforced in African countries, from pre-market testing and approvals to post-market surveillance of devices while in use. Such weak regulation and oversight may contribute to poor-quality and unsafe medical devices entering health systems, as reported in previous studies.^16 17 19 26^ In addition, there was a notable gap in guidance for donated devices, which continue to represent most of the medical devices introduced into African contexts.^15^ Guidelines for donation by the WHO and other donor partners were cited by a few studies in this review, however, there was limited information to indicate implementation and compliance.^34 38 43^ This finding reflects a previous study which also found limited information on the implementation of medical device donations in LMICs and poor compliance with WHO guidelines.^15^

### Implication of findings and proposed framework for future research

Taken together, regulatory and oversight processes identified in this review largely focus on the functions of regulatory agencies at the national government level. Furthermore, the literature only provided broad descriptions of medical device regulatory guidelines and processes, with limited information on how these may have been implemented in practice at national and subnational levels. Regulation and oversight at the national level through the function of NRAs remains a vital part of medical device governance. However, as shown in the review findings, such processes alone cannot adequately address the challenges of safety, quality and poor oversight of medical devices. These challenges are known to extend beyond regulating market introduction.^2 17 18 20^ Studies in this review and previous literature also highlight that in the absence of adequate formal governance processes, less formal to informal processes and relations become increasingly important and operate across various levels of the health system.^9 18 39 42^

Hence, the review findings highlight a need to further understand how countries implement governance arrangements (including at the subnational and health facility level), and how local processes interact with national/international policies and guidelines to shape governance and oversight of medical devices. This calls for the development of comprehensive frameworks which draw attention towards oversight beyond market authorisation. Such frameworks should be developed drawing on the rich insights and experiences of the various stakeholders involved in the governance of medical devices.^15 19^

**Figure 2:**
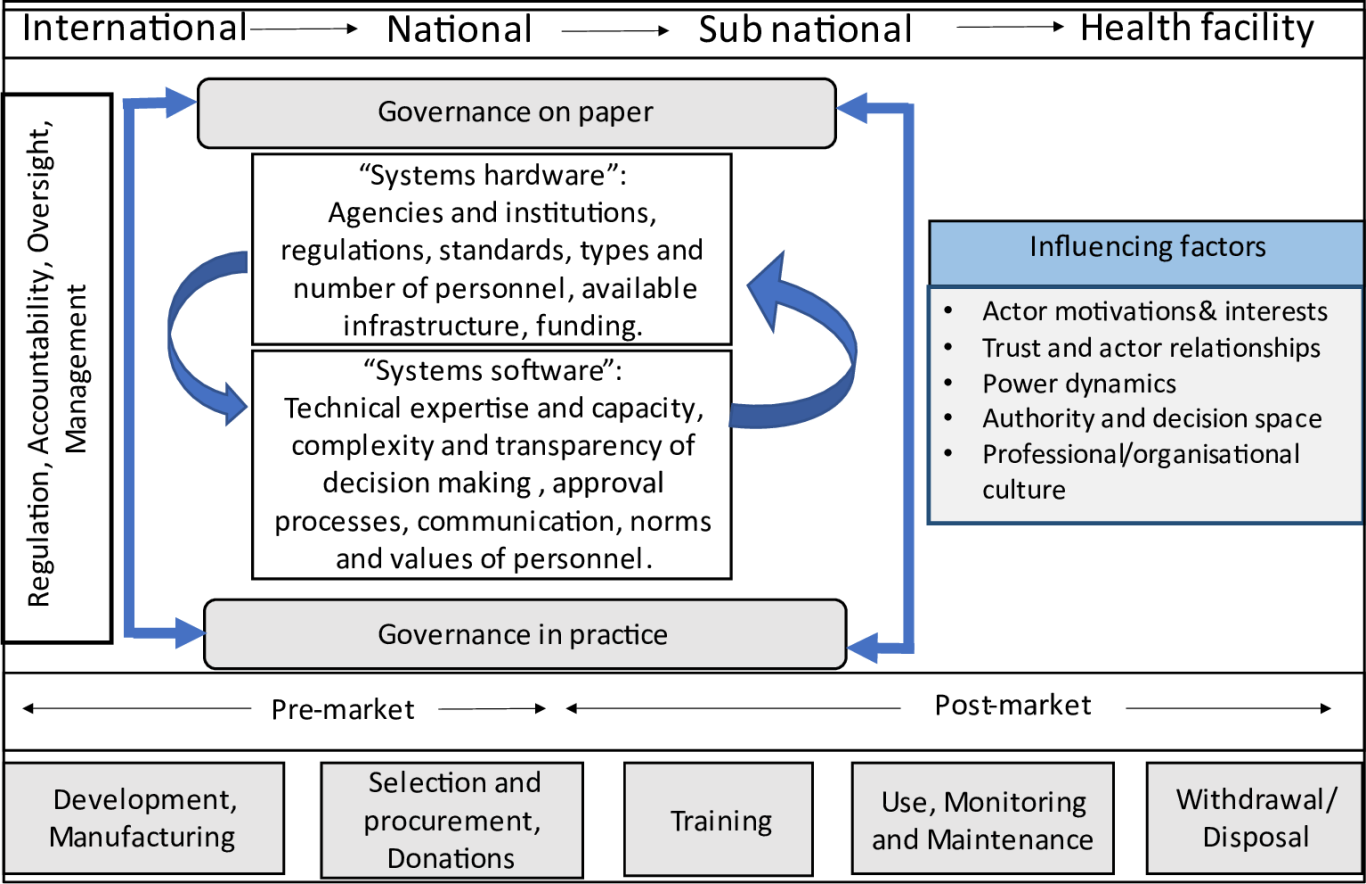
Conceptual framework for the examination of governance arrangements for medical devices.

To this end, we propose an initial conceptual framework (figure 2) to feed into efforts to examine governance arrangements for medical devices. Our framework draws on existing health system and health system governance frameworks and literature.^5 7–9 59^ It illustrates that the introduction and use of medical devices involves governance arrangements at various levels of the health system (including international, regional, national, subnational and health facility levels), and a wide range of actors and stakeholders within and across these levels. In practice, governance arrangements for medical devices span both ‘formal’ organisational structures, rules and processes (such as national regulatory agencies and agreed guidelines) as well as ‘informal’ dimensions (such as facility level processes and workarounds implemented to cope with resource constraints). Thus this framework incorporates elements that might be described as the ‘hardware’ (infrastructure, policies) of health systems, as well ‘tangible software’ (knowledge, skills and processes of decision-making) and intangible ‘software’ (relationships, interests, values and norms).^5^

Key functions of governance of medical devices such as regulation, accountability, oversight and management across the medical device lifecycle are enacted through the interplay of formal and informal rules and processes; and hardware and software elements. Influencing the nature of this interplay are wider system dynamics including power relations between actors, their authority and decision-making space and professional and organisational cultures. Given this complexity, the importance is underscored, of examining governance as intended on paper and governance as implemented in practice across all levels of health systems and seeking to understand the reasons for and implications of differences. Illustrations of research questions which have been overlooked which the framework points to include:

- Who are the actors involved in medical device governance processes (eg, regulation) at subnational and facility levels on paper and in practice and how might they interact?
- What factors (organisational and wider contextual) influence the everyday realities of how governance and oversight rules and processes are implemented?
- What are the implications of gaps between formal rules on paper and governance in practice for the regulation of medical devices across the health system?

We suggest this initial framework is a potentially valuable tool to inform future research on medical device governance and oversight, in particular encouraging greater emphasis of governance realities at national and subnational levels. This work, and development of more detailed versions of elements of the framework, should encourage further studies on relatively neglected areas of research. This will allow exploration of similarities and differences across contexts and support identification of potential shared (as well as context specific) points of intervention to strengthen governance. In these ways, we hope the framework will ultimately contribute to transferable lessons on how governance rules and processes might be improved to ensure the benefits of medical devices are achieved and harms are minimised.

### Strengths and limitations

This review employed a comprehensive, systematic search strategy identifying and mapping relevant peer-reviewed and grey literature on medical device regulation and oversight. It thus builds and expands on the knowledge from previous literature on medical device governance. However, there are some limitations. The search strategy aimed at being broad and inclusive but given the broad nature of the concepts of ‘governance’ and ‘medical device’, it is possible some relevant sources of evidence on topics related to these terms may have been excluded. Although no language limitations were specified to mitigate excluding relevant literature from non-English speaking parts of Africa, we recognise potentially relevant literature from such regions may have been missed.

## Conclusion

This study provided a broad overview of the governance, including regulation and oversight of medical devices within the African context and highlights gaps in the literature and challenges of medical device governance within this context. Current frameworks and guidelines are focused mainly on national-level guidelines and processes, which are generally described as inadequate. There is also limited information on the implementation of these national frameworks or what rules and processes apply beyond this level. Given the limited availability of empirical research on medical device governance, there is a need for future studies to examine governance arrangements carefully and elucidate where current guidance might be improved. We propose a conceptual framework in this paper to guide future more in-depth research in this regard.

## Data Availability

All data relevant to the study are included in the article or uploaded as supplementary information. Our search strategy is available in the supplemental files and the details of data extracted from the included literature are available in the supplemental files.
