# Supplementary material for: Medical device regulation and oversight in African countries: a scoping review of literature and development of a conceptual framework
Source: BMJ Glob Health. 2023 Aug 9;8(8):e012308. doi: 10.1136/bmjgh-2023-012308 (PMC10414093; doi:10.1136/bmjgh-2023-012308)
Supplement: Supplementary data [file bmjgh-2023-012308supp005.pdf]

Title: Medical device regulation and oversight at the national and sub-national level in sub-Saharan Africa: A scoping review protocol.

Background/introduction

Health technologies have shown potential to improve the quality, safety and outcomes of healthcare delivery[1,2]. However, the introduction, adoption and use of health technologies can be challenging in many settings, leading to poor adoption, difficulty in use and even unintended harms and risks[2,3]. Governance arrangements-including regulatory and oversight frameworks, systems and processes, can influence the safety, quality and use of health technologies which ultimately improves health outcomes[4]. Thus, regulatory and other governance arrangements are needed to ensure health technologies are prioritised, used safely, effectively, to maximise benefits and minimise risks[1,2].

Governance arrangements for health technologies are likely to be influenced by the way in which the technology is defined or categorised. Medical devices are noted in particular, as challenges with definitions and classification have been shown to influence regulation and oversight[5,6]. Broadly, medical devices are defined as health technologies other than medicines, vaccines, or clinical procedures, and include a wide range of products from simple medical supplies to more complex equipment[7,8]. In low and middle income countries, the regulation of medical devices is generally less well established than for other health technologies such as medicines and pharmaceuticals, with varying guidelines and processes applied[6,9–11].

Concerns about the governance of medical devices continue to be raised, made more pertinent by the significant numbers of devices and equipment found to be inappropriate, unsafe, non-functional, obsolete or abandoned[12–15]. Medical devices donated and procured for use are often designed in and for high income settings and may not be appropriate for low-resource settings[13,14]. Safety issues and other concerns are highlighted, including harm or risks to patients, inappropriate use by healthcare providers as well as the unavailability of expertise to repair and maintain equipment[2,14]. In addressing such concerns, governance and regulation has largely focused on identifying and addressing problems associated with device safety and its users[2]. But, systems issues such as accountability and reporting mechanisms, administrative strategies and organisational policies have been shown to influence safety and use concerns related to medical devices[16,17].

Reports show sub-Saharan Africa countries, continue to experience challenges with the development, availability and affordability of essential medical devices[6,10,14]. Inadequate and poorly understood regulatory and governance pathways for medical devices are highlighted as a key barrier to access, appropriate use and improved quality of care[10,18,19]. Where regulatory frameworks exist, these are often modelled after guidelines used by the US FDA, EU and other high-income settings[20]. Such guidelines from high income settings are seen to focus mainly on the approval of medical devices for market introduction, and it is unclear how these processes allow for oversight of medical devices beyond introduction into markets[10]. Also, the suitability of these regulatory processes for SSA contexts, for enabling the prioritisation of country-specific issues have been questioned[10].

Nasir N, *et al. BMJ Glob Health* 2023; 8:e012308. doi: 10.1136/bmjgh-2023-012308

A preliminary search in MEDLINE, PubMed and Scopus returned a few literature reviews of medical device regulation in select African countries[20,21]. The literature shows only a small number of countries in SSA have developed specific guidelines and processes for the regulation of medical devices[10,20,21]. National frameworks and systems for regulation of medical devices were discussed mainly by comparison with the World Health Organization (WHO) guidelines, although other international regulatory guidelines such as the US FDA, International Standards Organisation (ISO) and the EU "*conformité européenne*" (CE) process were recognised[20,21].

Overall, it is not well understood how medical device regulation and oversight does (or should) take place within sub-Saharan Africa; and there is limited evidence of the implementation of regulatory and oversight processes across national and sub-national levels of governance. Furthermore, it is unclear how medical devices introduced into the market are adopted by healthcare organisations such as hospitals and other health facilities, and what governance processes may be applicable. This study will explore the nature and extent of information available to understand how medical devices are regulated and overseen in sub-Saharan Africa, examining processes both at national and sub-national level and explore the evidence of the implementation of current approaches in practice.

Protocol design

The scoping review will be conducted in accordance with the guidance provided by the Joanna Briggs Institute Manual for Evidence Synthesis[22]. This builds on earlier guidance on scoping reviews provided by Arksey and O'Malley[23], Levac and colleagues[24] and Peters et al[25], providing an updated scoping review methodology. In addition, guidance on the conduct and reporting of scoping reviews as outlined in the PRISMA-ScR guidelines will be followed[26].

Aim and Review Question(s)

The overall aim of the systematic scoping review is to explore the literature on how medical devices are regulated and overseen at national and sub-national level in sub-Saharan Africa. The review questions include the following:

1. How are medical devices categorised and defined in the literature for the purposes of regulation and oversight?
2. What regulations/policies and guidance are in place and does this differ by type/classification of medical device?
3. Who are the actors involved in the governance (including regulation and oversight) of medical devices?
4. How are governance processes implemented in practice at the national and sub-national levels- including challenges and opportunities for improving regulatory and oversight guidelines and processes.

Eligibility criteria

This study aims to describe how medical device regulation and oversight occurs in SSA. This review will build upon previous non-systematic literature reviews outlining regulatory frameworks in select countries. It will also include a wide range of resources (published and grey literature) which describe or report how medical device regulation and oversight policies

and guidelines work and experiences implementing these processes across national and sub-national levels in SSA countries.

Population

This review will include resources on regulatory and oversight guidelines and processes for medical devices used in all populations/types of participants.

Concept

The concept of this review is the governance- including regulation and oversight of medical devices. The review will include resources which either focus on or include information on regulation and other governance processes at the national and subnational level.

Context

The broad context of the papers included in this review is sub-Saharan Africa.

Types of sources for inclusion:

This will include scientific peer-reviewed studies, policies/frameworks and guidelines available, dissertations/thesis, any commentaries on policies, and empirical papers reporting experience implementing policies/guidelines. Literature on regulation of medical devices outside the sub-Saharan African context may be included where it provides information relevant to medical device regulation and oversight in SSA, such as the WHO and other global guidelines.

Exclusion criteria: Literature detailing regulation and oversight of medicinal products, medicines or pharmaceuticals, vaccines and any health technology that is not a medical device will be excluded except where there is overlapping relevant information. Articles focused on evaluation or testing of specific medical devices for efficacy, safety, or quality in human or animal populations will be excluded. Literature only providing details of a specific country's Act or legislation for medical regulation will be excluded.

Search Strategy and information sources

The information sources in this systematic scoping review will include databases such as PubMed, MEDLINE, Scopus, EMBASE, Web of Science, CINAHL, African Journals Online, African Digital Archive, Policy Commons, and the websites of key organisations involved in medical device regulation such as the World Health Organisation (WHO).

First, an initial search will be conducted to explore terms and keywords for the searches. Second, the identified terms and keywords will be applied to searches across the chosen databases, and the results will be organised. Third, hand searching of all reference lists of included studies to identify additional material of relevance. Next, a search of a variety of grey literature sources such as relevant grey literature databases (eg, Grey Literature Report, OpenGrey, Web of Science Conference Proceedings) will be done to identify studies, reports of relevance to this review. Finally, a targeted search of websites of relevant organisations will be conducted to reveal additional material not found in the database search.

Nasir N, *et al. BMJ Glob Health* 2023; 8:e012308. doi: 10.1136/bmjgh-2023-012308

Key terms for the review include "medical devices", "regulation", "governance", "oversight", "sub-Saharan Africa" and their synonyms. Key words for the search in PubMed are shown below as an example. Terms will be searched as both keywords in the title and/or abstract and subject headings (eg, MeSH) as appropriate. No language or date limits will be applied.

Search terms and key words for PubMed:

(((("Equipment and Supplies"[Mesh])) OR ("medical device*"[Title/Abstract])) AND (("Africa South of the Sahara"[Mesh]) OR (("Subsaharan Africa"[Text Word] or "Sub-Saharan Africa"[Text Word] or Angola or Benin or Botswana or "Burkina Faso"[Text Word] or Burundi or "Cabo Verdi"[Text Word] or Cameroon or "Cape Verde"[Text Word] or "Central African Republic"[Text Word] or Chad or Comoros or Congo or "Cote d'Ivoire"[Text Word] or Djibouti or "Equatorial Guinea"[Text Word] or Eritrea or Eswatini or Ethiopia or Gabon or Gambia or Ghana or Guinea or Guinea-Bissau or Kenya or Lesotho or Liberia or Madagascar or Malawi or Mali or Mauritania or Mauritius or Mozambique or Namibia or Niger or Nigeria or Reunion or Rwanda or "Sao Tome"[Text Word] or Principe or Senegal or Seychelles or "Sierra Leone"[Text Word] or Somalia or "South Africa"[Text Word] or Sudan or Swaziland or Tanzania or Togo or Uganda or "Western Sahara"[Text Word] or Zambia or Zimbabwe) OR ("east africa*"[Text Word] OR "central africa*"[Text Word] OR "southern africa*"[Text Word])))) AND ((regulation[Title/Abstract] OR oversight[Title/Abstract] OR governance[Title/Abstract] OR "regulatory framework*"[Title/Abstract] OR "regulatory establishment"[Title/Abstract] OR "regulatory process*"[Title/Abstract] OR "regulatory authorit*"[Title/Abstract] OR "regulatory capacity"[Title/Abstract]) OR (("Device Approval"[Mesh]) OR "Government Regulation"[Mesh]))

Study selection

The Covidence online software platform will be used to facilitate the entire screening process. Mendeley Reference Management software will be used to collect and upload all identified citations. Duplicates will be removed. Titles and abstracts of the papers of current interest will then be assessed independently by 2 reviewers against the eligibility criteria outline above. Potential full text papers for inclusion will undergo the same procedure, being assessed in detail against the inclusion criteria. Reasons for exclusion of full text studies will be recorded and reported. Any disagreements between the 2 reviewers will be resolved by discussion to reach consensus or the involvement of an additional reviewer where necessary.

Data collection/charting

A data extraction tool will be developed to extract study characteristics and relevant details about the population, concept, context, and key findings. The data extraction tool may be modified and revised during the data charting process and eventual changes will be described in the final report. Study characteristics to be extracted will include, but not be limited to: publication year, publication type, country/region of focus, actors, available regulatory frameworks; medical device definition; device classification; pre-market controls; placing-on-market controls; post-market controls; challenges with regulation and oversight and opportunities to improve regulation and oversight. These categories are based on previous literature[21,27]. The first reviewer will extract the data and a proportion of this will be independently checked by the second reviewer.

Data summary and synthesis

Data will be summarised to align with the aim and research questions of the systematic scoping review. This will include the use of tables and charts, along with a narrative summary. In an effort to understand the governance roles of various health system actors in the regulation and oversight of medical devices, this review will apply the multi-level governance framework[28,29] to guide the analysis of findings.

Stakeholder consultation

This scoping review may include consultation with relevant stakeholders and content experts following the initial search and study selection process to refine the search strategy and identify any key missing resources. Stakeholder may also be consulted to further explore the findings, which may provide insights beyond what is reported in the literature.

Discussion

The results from this scoping review will aim to understand the current state of medical device regulation and oversight in sub-Saharan Africa, explore challenges and opportunities for governance of medical devices and help to identify strengths and weakness of the approaches to medical device regulation in these settings.

Conclusion

Medical devices are widely used in sub-Saharan Africa to improve a variety of health conditions. However, their introduction and use has been associated with harms and risks to the public and health systems. Regulatory, oversight and other governance systems are needed to ensure safety, quality and effectiveness but these remain limited and not well understood. This scoping review aims to map the literature available, clarify key concepts and identify important roles and issues related to governance processes for medical devices.

References

1. Perakslis E, Ginsburg S G. Digital Health- The Need to Assess Benefits, Risks and Value. JAMA. 2021;325(2):127–8.

2. Mytton OT, Velazquez A, Banken R, Mathew JL, Ikonen TS, Taylor K, et al. Introducing new technology safely. Qual Saf Health Care [Internet]. 2010 [cited 2021 Apr 19];19 Suppl 2. Available from: http://www.ghtf.org

3. Greenhalgh T, Wherton J, Papoutsi C, Lynch J, Hughes G, A'Court C, et al. Beyond adoption: A new framework for theorizing and evaluating nonadoption, abandonment, and challenges to the scale-up, spread, and sustainability of health and care technologies. J Med Internet Res [Internet]. 2017 [cited 2021 Jan 25];19(11). Available from: http://www.jmir.org/2017/11/e367/

4. Herrera CA, Lewin S, Paulsen E, Ciapponi A, Opiyo N, Pantoja T, et al. Governance arrangements for health systems in low-income countries: An overview of systematic

reviews. Cochrane Database Syst Rev [Internet]. 2017 [cited 2021 Apr 18];2017(9). Available from: www.cochranelibrary.com

5. Usang UE, Inyang AW, Ilori I-AU, Inyama-Asuquo M, U.E. U, A.W. I, et al. Utilization of banked blood in pediatric surgical procedures in Calabar, Nigeria. African J Paediatr Surg [Internet]. 2013;10(2):145–9. Available from: http://ovidsp.ovid.com/ovidweb.cgi?T=JS&PAGE=reference&D=emed14&NEWS=N&AN=369694858

6. Aronson JK, Heneghan C, Ferner RE. Medical Devices: Definition, Classification, and Regulatory Implications. Drug Saf [Internet]. 2020 [cited 2021 May 2];43(2):83–93. Available from: https://doi.org/10.1007/s40264-019-00878-3

7. WHO Regional Office for Europe. Health technologies [Internet]. World Health Organization; 2021 [cited 2021 Nov 11]. Available from: https://www.euro.who.int/en/health-topics/Health-systems/health-technologies-and-medicines/health-technologies

8. World Health Organization (WHO). MEDICAL DEVICES [Internet]. 2021 [cited 2021 Nov 12]. Available from: https://www.who.int/health-topics/medical-devices#tab=tab_1

9. Cheng M. Medical device regulations: Global overview and guiding principles. [Internet]. Vol. 24. 2003 [cited 2021 May 1]. Available from: www.who.int/bct

10. WHO. MEDICAL DEVICES : Managing the mismatch: an outcome of the priority medical devices project. 2010 [cited 2021 Jun 30];1–36. Available from: https://apps.who.int/iris/bitstream/handle/10665/44407/9789241564045_eng.pdf

11. Henshall C, Mardhani-Bayne L, Frønsdal KB, Klemp M. Interactions between health technology assessment, coverage, and regulatory processes: Emerging issues, goals, and opportunities On behalf of the HTAi Policy Forum. Int J Technol Assess Health Care [Internet]. 2021 [cited 2021 May 2];27:253–60. Available from: https://www.cambridge.org/core/terms.https://doi.org/10.1017/S0266462311000262DownloadedfromHttps://www.cambridge.org/core.

12. Perry L, Malkin R. Effectiveness of medical equipment donations to improve health systems: How much medical equipment is broken in the developing world? Med Biol Eng Comput [Internet]. 2011 Jul 20 [cited 2021 Jun 30];49(7):719–22. Available from: https://link.springer.com/article/10.1007/s11517-011-0786-3

13. McDonald S, Fabbri A, Parker L, Williams J, Bero L. Medical donations are not always free: an assessment of compliance of medicine and medical device donations with World Health Organization guidelines (2009–2017). Int Health [Internet]. 2019 Sep 2 [cited 2021 Aug 18];11(5):379–402. Available from: https://academic.oup.com/inthealth/article/11/5/379/5420717

14. Ayah R, Ong'ech J, Mbugua EM, Kosgei RC, Waller K, Gathara D. Responding to maternal, neonatal and child health equipment needs in Kenya: a model for an innovation ecosystem leveraging on collaborations and partnerships. BMJ Innov [Internet]. 2020 Jul 1 [cited 2021 Aug 3];6(3):85–91. Available from: https://innovations.bmj.com/content/6/3/85

15. Charlesworth M, Zundert AAJ van. Medical device regulation: the need for clinical vigilance and oversight. Anaesthesia [Internet]. 2019 Jun 1 [cited 2021 Jul

16];74(6):693–5. Available from: https://ezproxy-prd.bodleian.ox.ac.uk:6493/doi/full/10.1111/anae.14603

16. Balka E, Doyle-Waters M, Lecznarowicz D, FitzGerald JM. Technology, governance and patient safety: Systems issues in technology and patient safety. Int J Med Inform. 2007;76(SUPPL. 1):S35–47.

17. Williams DB, Austin Z, Kohler JC, Howard A, Cheng Dinsie B; ORCID: http://orcid.org/0000-0002-6423-5242 Y-LAO-W. A framework for the management of donated medical devices based on perspectives of frontline public health care staff in Ghana. Med Access Point Care [Internet]. 2020;4. Available from: https://journals.sagepub.com/doi/full/10.1177/2399202620941367

18. Ginsburg AS, Macharia W, Ansermino JM. Evaluating neonatal medical devices in Africa. Lancet Digit Heal [Internet]. 2021 Apr 1 [cited 2021 Jul 11];3(4):e211–2. Available from: http://www.thelancet.com/article/S258975002100025X/fulltext

19. Lingg M, Mansilla AD, Durán-Arenas L, Wyss K. The regulation, assessment, and management of medical devices in Mexico: How do they shape the quality of delivered healthcare? Saf Heal [Internet]. 2017 Mar 14 [cited 2021 Nov 11];3(1):1–10. Available from: https://safetyinhealth.biomedcentral.com/articles/10.1186/s40886-017-0055-8

20. Hubner S, Maloney C, Phillips SD, Doshi P, Mugaga J, Ssekitoleko RT, et al. The Evolving Landscape of Medical Device Regulation in East, Central, and Southern Africa. Glob Heal Sci Pract [Internet]. 2021 Mar 24 [cited 2021 Mar 25];9(1):1–13. Available from: http://www.ghspjournal.org/lookup/doi/10.9745/GHSP-D-20-00578

21. Rugera SPP ete. P, McNerney R, Poon AK, Akimana G, Mariki RFF orge., Kajumbula H, et al. Regulation of medical diagnostics and medical devices in the East African community partner states. BMC Health Serv Res [Internet]. 2014 Dec 31 [cited 2021 Jun 30];14(1):524. Available from: https://bmchealthservres.biomedcentral.com/articles/10.1186/s12913-014-0524-2

22. Peters M, Godfrey C, McInerney P, Munn Z, Tricco AC, Khalil H. JBI Manual for Evidence Synthesis. In: Aromataris E, Munn Z, editors. JBI Manual for Evidence Synthesis [Internet]. The Joanna Briggs Institute JBI; 2020. p. 487. Available from: https://synthesismanual.jbi.global

23. Arksey H, O'Malley L. Scoping studies: Towards a methodological framework. Int J Soc Res Methodol Theory Pract [Internet]. 2005 Feb [cited 2021 Jun 30];8(1):19–32. Available from: https://www.tandfonline.com/doi/abs/10.1080/1364557032000119616

24. Levac D, Colquhoun H, O'Brien KK. Scoping studies: Advancing the methodology. Implement Sci [Internet]. 2010 Sep 20 [cited 2021 Jun 30];5(1):1–9. Available from: http://www.cihr-irsc.ca

25. Peters MDJ, Marnie C, Tricco AC, Pollock D, Munn Z, Alexander L, et al. Updated methodological guidance for the conduct of scoping reviews. JBI Evid Synth [Internet]. 2020 Oct 1 [cited 2021 Jun 30];18(10):2119–26. Available from: https://rgu-repository.worktribe.com/output/979072/updated-methodological-guidance-for-the-conduct-of-scoping-reviews

26. Tricco AC, Lillie E, Zarin W, O KK, Colquhoun H, Levac D, et al. PRISMA Extension for Scoping Reviews (PRISMA-ScR): Checklist and Explanation. Ann

Intern Med. 2018;169:467–73.

27. World Health Organization (WHO). Global Model Regulatory Framework for Medical Devices including in vitro diagnostic medical devices. [Internet]. WHO Medical device technical series. Licence: CC BY-NC-SA 3.0 IGO. Geneva; 2017 [cited 2021 Jul 8]. Available from: http://apps.who.int/bookorders.

28. Abimbola S, Negin J, Jan S, Martiniuk A. Towards people-centred health systems: a multi-level framework for analysing primary health care governance in low- and middle-income countries. Health Policy Plan [Internet]. 2014 Sep 1 [cited 2021 Jul 12];29(suppl_2):ii29–39. Available from: https://academic.oup.com/heapol/article/29/suppl_2/ii29/585925

29. Pyone T, Smith H, Van Den Broek N. Frameworks to assess health systems governance: A systematic review. Health Policy Plan. 2017;32(5):710–22.
