# Supplementary material for: Medical device regulation and oversight in African countries: a scoping review of literature and development of a conceptual framework
Source: BMJ Glob Health. 2023 Aug 9;8(8):e012308. doi: 10.1136/bmjgh-2023-012308 (PMC10414093; doi:10.1136/bmjgh-2023-012308)
Supplement: Supplementary data [file bmjgh-2023-012308supp004.pdf]

PubMed 2nd July: updated 2/08/21: 148 results.

(((("Equipment and Supplies"[Mesh])) OR ("medical device*"[Title/Abstract])) AND (("Africa South of the Sahara"[Mesh]) OR (("Subsaharan Africa"[Text Word] or "Sub-Saharan Africa"[Text Word] or Angola or Benin or Botswana or "Burkina Faso"[Text Word] or Burundi or "Cabo Verdi"[Text Word] or Cameroon or "Cape Verde"[Text Word] or "Central African Republic"[Text Word] or Chad or Comoros or Congo or "Cote d'Ivoire"[Text Word] or Djibouti or "Equatorial Guinea"[Text Word] or Eritrea or Eswatini or Ethiopia or Gabon or Gambia or Ghana or Guinea or Guinea-Bissau or Kenya or Lesotho or Liberia or Madagascar or Malawi or Mali or Mauritania or Mauritius or Mozambique or Namibia or Niger or Nigeria or Reunion or Rwanda or "Sao Tome"[Text Word] or Principe or Senegal or Seychelles or "Sierra Leone"[Text Word] or Somalia or "South Africa"[Text Word] or Sudan or Swaziland or Tanzania or Togo or Uganda or "Western Sahara"[Text Word] or Zambia or Zimbabwe) OR ("east africa*"[Text Word] OR "central africa*"[Text Word] OR "southern africa*"[Text Word])))) AND ((regulation[Title/Abstract] OR oversight[Title/Abstract] OR governance[Title/Abstract] OR "regulatory framework*"[Title/Abstract] OR "regulatory establishment"[Title/Abstract] OR "regulatory process*"[Title/Abstract] OR "regulatory authorit*"[Title/Abstract] OR "regulatory capacity"[Title/Abstract]) OR (("Device Approval"[Mesh]) OR "Government Regulation"[Mesh]))

Embase (Ovid) 2nd July 2021 ,updated 2nd August 2021: 69 results

1. "diagnostic device*" OR "medical device*"
2. "Subsaharan Africa" or "Sub-Saharan Africa" or “Africa” or Angola or Benin or Botswana or "Burkina Faso" or Burundi or "Cabo Verdi" or Cameroon or "Cape Verde" or "Central African Republic" or Chad or Comoros or Congo or "Cote d'Ivoire" or Djibouti or "Equatorial Guinea" or Eritrea or Eswatini or Ethiopia or Gabon or Gambia or Ghana or Guinea or Guinea-Bissau or Kenya or Lesotho or Liberia or Madagascar or Malawi or Mali or Mauritania or Mauritius or Mozambique or Namibia or Niger or Nigeria or Reunion or Rwanda or "Sao Tome" or Principe or Senegal or Seychelles or "Sierra Leone" or Somalia or "South Africa" or Sudan or Swaziland or Tanzania or Togo or Uganda or "Western Sahara" or Zambia or Zimbabwe OR "east africa*" OR "central africa*" OR "southern africa*"
3. regulation OR oversight OR governance OR "regulatory" OR “registration” OR "postmarket*" OR "post-market*" OR "Device Approval"
4. 1 AND 2 and 3.

Scopus 15th July 12.29pm 83 results, updated 2nd August

("diagnostic device*" OR "medical device*" OR "medical equipment") AND TITLE-ABS-KEY (("Subsaharan Africa" OR "Sub-Saharan Africa" OR africa OR angola OR benin OR botswana OR "Burkina Faso" OR burundi OR "Cabo Verdi" OR cameroon OR "Cape Verde" OR "Central African Republic" OR chad OR comoros OR congo OR "Cote d'Ivoire" OR djibouti OR "Equatorial Guinea" OR eritrea OR eswatini OR ethiopia OR gabon OR gambia OR ghana OR guinea OR guinea-bissau OR

kenya OR lesotho OR liberia OR madagascar OR malawi OR mali OR mauritania OR mauritius OR mozambique OR namibia OR niger OR nigeria OR reunion OR rwanda OR "Sao Tome" OR principe OR senegal OR seychelles OR "Sierra Leone" OR somalia OR "South Africa" OR sudan OR swaziland OR tanzania OR togo OR uganda OR "Western Sahara" OR zambia OR zimbabwe OR "east africa*" OR "central africa*" OR "southern africa*")) AND TITLE-ABS-KEY ((regulation OR oversight OR governance OR stewardship OR "regulatory" OR "registration" OR "approval" OR "postmarket*" OR "post-market*" OR "Device Approval")))

Medline (Ovid) 15th July 2021. Updated 2nd August

Medline (Ovid MEDLINE® Epub Ahead of Print, In-Process & Other Non-Indexed Citations, Ovid MEDLINE® Daily and Ovid MEDLINE®) 1946 to present

1. ("diagnostic device*" or "medical device*" or "medical equipment").mp. [mp=title, abstract, original title, name of substance word, subject heading word, floating sub-heading word, keyword heading word, organism supplementary concept word, protocol supplementary concept word, rare disease supplementary concept word, unique identifier, synonyms]: 22348 results.

2. ("Subsaharan Africa" or "Sub-Saharan Africa" or "Africa" or Angola or Benin or Botswana or "Burkina Faso" or Burundi or "Cabo Verdi" or Cameroon or "Cape Verde" or "Central African Republic" or Chad or Comoros or Congo or "Cote d'Ivoire" or Djibouti or "Equatorial Guinea" or Eritrea or Eswatini or Ethiopia or Gabon or Gambia or Ghana or Guinea or Guinea-Bissau or Kenya or Lesotho or Liberia or Madagascar or Malawi or Mali or Mauritania or Mauritius or Mozambique or Namibia or Niger or Nigeria or Reunion or Rwanda or "Sao Tome" or Principe or Senegal or Seychelles or "Sierra Leone" or Somalia or "South Africa" or Sudan or Swaziland or Tanzania or Togo or Uganda or "Western Sahara" or Zambia or Zimbabwe or "east africa*" or "central africa*" or "southern africa*").mp. [mp=title, abstract, original title, name of substance word, subject heading word, floating sub-heading word, keyword heading word, organism supplementary concept word, protocol supplementary concept word, rare disease supplementary concept word, unique identifier, synonyms] 533356 results.
3. (regulation or oversight or governance or stewardship or "regulatory" or "registration" or "postmarket*" or "post-market*" or "approval" or "Device Approval").mp. [mp=title, abstract, original title, name of substance word, subject heading word, floating sub-heading word, keyword heading word, organism supplementary concept word, protocol supplementary concept word, rare disease supplementary concept word, unique identifier, synonyms]: 2036707 results.

4. 1 and 2 and 3: 36 results

Web of Science 15th July 22.39pm: Web of Science Core Collection, BIOSIS Citation Index, Current Contents Connect, Data Citation Index, MEDLINE®, SciELO Citation Index: updated 2nd August 2021.

((TS=(("diagnostic device*" or "medical device*" or "medical equipment"))) AND TS=(("Subsaharan Africa" or "Sub-Saharan Africa" or "Africa" AND TS=((regulation or oversight or governance or stewardship or "regulatory" or "registration" or "postmarket*" or "post-market*" or "approval" or "Device Approval"))

CINAHL (EBSCohost) 2nd August 2021 32 results

1. S1 "(MM "Medical Device Legislation") OR (MM "Equipment and Supplies+") OR (MH "Product Surveillance") OR "medical devices or medical equipment or medical technology"
2. S2 "Subsaharan Africa" or "Sub-Saharan Africa" or "Africa"
3. S3 (medical dev regulation or oversight or governance or stewardship or "regulatory" or "registration" or "postmarket*" or "post-market*" or "approval" or "Device Approval"
4. S1 AND S2 AND S3.

Science Direct 15th July 2021
“medical device” AND regulation OR Oversight AND Africa
296 results. No relevant documents.

Google Scholar search 5-6th August 2021. 1st 30 pages of results
Search terms adapted to: "medical device*" regulation OR oversight OR governance africa OR sub-saharan Africa

Google search on 2nd July 2021.
"medical device *" site:.who.int filetype:pdf to find WHO publications.

11th July 2021: Searched the **WHO website**: https://www.who.int/publications/i for medical device. Also the following non indexed data bases searched: African Journals Online, African Digital Archive, Policy Commons.
