# Supplementary material for: Medical device regulation and oversight in African countries: a scoping review of literature and development of a conceptual framework
Source: BMJ Glob Health. 2023 Aug 9;8(8):e012308. doi: 10.1136/bmjgh-2023-012308 (PMC10414093; doi:10.1136/bmjgh-2023-012308)
Supplement: Supplementary data [file bmjgh-2023-012308supp001.pdf]

Table S1: Characteristics of included sources of evidence.

Author/Year	Title of Publication/Document	Type of publication/document	Country/Region of focus	Objective of the document
DeMaria et al (2018)[34]	Safe innovation: On medical device legislation in Europe and Africa	Journal article *Health Policy and Technology*	Africa: 8 countries including Kenya, Egypt, Malawi, Tanzania, Uganda, Nigeria, SA, Ethiopia.	To compare the certification route that manufacturers have to respect for marketing a medical device in some African countries. Several African countries have oriented their regulatory processes for medical devices on the EU system. In this paper, after outlining the EU regulatory framework, we will analyse the regulatory landscape in a number of African countries with at least a representative of the African Biomedical Engineering Consortium (ABEC)
Dacombe et al (2019)[35]	Regulation of HIV self-testing in Malawi, Zambia and Zimbabwe: a qualitative study with key stakeholders.	Journal article *Journal of the International AIDS Society*	Malawi, Zambia and Zimbabwe	To determine the current regulatory status of HIV self-test kits in Malawi, Zambia and Zimbabwe and document the perceptions and suggestions of key stakeholders regarding current and future HIVST regulation in each country.

Dube-Mwezi et al (2020)[36]	A rapid assessment of the National Regulatory Systems for medical products in the Southern African Development Community	Journal article<br>*Journal of Pharmaceutical Policy and Practice*	South African Development Community	Analysing the regulatory systems for medical products in the 16 Member States of the Southern African Development Community (SADC). Themes such as the governance, regulatory framework for medical products, and medical devices and equipment framework were explored.
Hubner et al (2021)[37]	The Evolving Landscape of Medical Device Regulation in East, Central, and Southern Africa	Journal article<br>*Global Health Science and Practice*	14 member countries of the College of Surgeons of East, Central, and Southern Africa, and South Africa (COSECA): Botswana, Burundi, Ethiopia, Kenya, Malawi, Mozambique, Namibia, Rwanda, South Sudan, Sudan, Tanzania, Uganda, Zambia and Zimbabwe.	The review provides a summary of the state of medical device regulation in the countries of focus.
Kedwani et al (2019)[38]	Analysis of regulatory requirements of medical devices and in-vitro diagnostics worldwide for the development of an efficient procedure of registration for manufacturers of medical products	Journal article<br>*Current Directions in Biomedical Engineering*	Global (including Africa)	The paper investigates the role and activities of the most important harmonization groups in the design of the global regulatory landscape for MDs and IVDs
McNerny and Peeling (2015)[39]	Regulatory In Vitro Diagnostics Landscape in Africa: Update on Regional Activities	Journal article<br>*Clinical Infectious Diseases*	Africa	The article reviews the regulation of diagnostic tests for tuberculosis case detection with a focus on the World Health Organization (WHO) AFRO region

McNerny et al (2014)[40]	Improving access to new diagnostics through harmonised regulation: priorities for action	Journal article *Afr J Lab Med*	Developing countries (including in Africa)	To provide an overview of regulation of diagnostic tests in developing countries.
Mori et al (2011)[41]	Quality of medical devices and in vitro diagnostics in resource-limited settings	Journal article *Tropical medicine and International Health*	Resource limited settings/Global South countries	The paper describes evidence of quality problems of MDs or IVDs in resource-limited settings and regulatory challenges obtained through literature review, observations and surveys.
Wong et al (2014)[42]	HIV self-testing in resource-limited settings: Regulatory and policy considerations	Journal article *AIDS and Behavior*	Resource limited settings	To examine regulatory and policy issues with HIVST, and similar self-diagnostic devices.
Rugera et al (2014)[43]	Regulation of medical diagnostics and medical devices in the East African community partner states	Journal article *BMC Health Services Research*	East African Community (EAC) a regional inter- governmental organization of five Partner States: Kenya, Uganda, Burundi, Tanzania and Rwanda	To assess capacity and current practices across the Partner States for regulation of medical diagnostics (including In-vitro Diagnostics) and medical devices. Questions were framed to collect data on each of the essential features of a regulatory program for medical devices as described in guidelines published by the World Health Organisation

WHO (2020)[44]	Guidance for post-market surveillance and market surveillance of medical devices, including in vitro diagnostics	Guideline/report	Global	Describes objectives and processes for post-market surveillance for medical devices conducted by manufacturers with the assistance of their economic operators, as well as market surveillance conducted by regulators, and the role of other stakeholders in these processes.
World Health Organization (2003)[45]	Medical device regulations: Global overview and guiding principles.	Guideline/report	Global	To provide guidance to Member states to set up or modify national regulatory systems for medical devices.
WHO (2017)[46]	Global Model Regulatory Framework for Medical Devices including in vitro diagnostic medical devices.	Guideline/report	Global	Intended to provide guidance and support to WHO Member States that have yet to develop and implement regulatory controls relating to medical devices, as well as to jurisdictions that are continuing to improve their regulatory frameworks as they take steps to ensure the quality and safety of medical devices available in their countries.
WHO (2016)[47]	Medical device regulatory systems at country level	Report	Egypt	Brief description of the medical device regulatory system
WHO (2016)[48]	Medical device regulatory systems at country level	Report	Ethiopia	Brief description of the medical device regulatory system
WHO (2016)[49]	Medical device regulatory systems at country level	Report	Ghana	Brief description of the medical device regulatory system

WHO (2016)[50]	Medical device regulatory systems at country level	Report	Togo	Brief description of the medical device regulatory system
WHO (2016)[51]	Medical device regulatory systems at country level	Report	Nigeria	Brief description of the medical device regulatory system
WHO (2016)[52]	Medical device regulatory systems at country level	Report	Zimbabwe	Brief description of the medical device regulatory system
WHO (2016)[53]	Medical device regulatory systems at country level	Report	Libya	Brief description of the medical device regulatory system
WHO (2016)[54]	Medical device regulatory systems at country level	Report	Namibia	Brief description of the medical device regulatory system
WHO (2016)[55]	Medical device regulatory systems at country level	Report	Cabo Verde	Brief description of the medical device regulatory system
WHO (2016)[56]	Medical device regulatory systems at country level	Report	Tanzania	Brief description of the medical device regulatory system
WHO (2016)[57]	Medical device regulatory systems at country level	Report	Kenya	Brief description of the medical device regulatory system
WHO (2016)[58]	Medical device regulatory systems at country level	Report	Burkina Faso	Brief description of the medical device regulatory system
WHO (2016)[59]	Medical device regulatory systems at country level	Report	Algeria	Brief description of the medical device regulatory system

Peeling Rosana (2015)[60]	Update on Regional Harmonization of Diagnostic Regulation in Africa	Report	Africa	Update on harmonization efforts to improve regulatory oversight for diagnostic devices in Africa
WHO (2016)[61]	Medical device regulatory systems at country level	Report	South Africa	Brief description of the medical device regulatory system
WHO (2016)[62]	Medical device regulatory systems at country level	Report	Madagascar	Brief description of the medical device regulatory system
WHO (2016)[63]	Medical device regulatory systems at country level	Report	Sierra Leone	Brief description of the medical device regulatory system
WHO (2016)[64]	Medical device regulatory systems at country level	Report	Morocco	Brief description of the medical device regulatory system
WHO (2016)[65]	Medical device regulatory systems at country level	Report	Sudan	Brief description of the medical device regulatory system
WHO (2016)[66]	Medical device regulatory systems at country level	Report	Zambia	Brief description of the medical device regulatory system
WHO (2016)[67]	Medical device regulatory systems at country level	Report	Rwanda	Brief description of the medical device regulatory system
Lissel et al (2016)[68]	Conference proceedings: Annual International Conference of the IEEE Engineering in Medicine and Biology Society.	Conference report	African Biomedical Engineering Consortium (ABEC) partner countries.	This paper focuses on the development of an affordable and sustainable system for medical device regulations to provide safe, effective and quality healthcare products for Africa

Piaggio et al (2020)[69]	Donation of Medical Devices in Low-Income Countries: Preliminary Results from Field Studies	Conference reports IFMBE Proceedings 73	Sub-Saharan Africa	Undertake field studies in 3 sub-Saharan countries towards understanding some of the reasons behind the non-use or the breakage of the MDs.
Saidi and Douglas (2016)[70]	Medical device regulation in Africa	Book Chapter Biomedical Engineering For Africa	Africa	To provide an overview of international standards and regulations for medical devices focused on focus on the ten African countries with the highest GDP – Kenya, Nigeria, Egypt, Sudan, Morocco, Angola, Algeria, Tanzania, Ethiopia and South Africa
Dusabe Gloria (2020)[71]	*Doctoral disseration University of Malta*/Regulation of medical devices in Europe and Africa	Dissertation/thesis	Europe and Africa: Ghana, Kenya, Rwanda, Tanzania and Uganda	To evaluate the regulations and guidelines for medical devices in Europe (Germany and Switzerland) and five countries in Africa, namely, Uganda, Kenya, Tanzania, Rwanda and Ghana with the aim of identifying gaps and proposing strategies for improvement.
African Union Development Agency [AUDA-NEPAD] (2019)[72]	Africa Medical Devices Forum (AMDF)	Webpage	Africa	To establish a harmonized framework for regulation of medical devices including in vitro diagnostics in Africa based on the WHO global model for Medical Devices Regulatory Framework Model.

*Citation number as it appears on the manuscript reference list is in superscript.
