# Supplementary material for: Medical device regulation and oversight in African countries: a scoping review of literature and development of a conceptual framework
Source: BMJ Glob Health. 2023 Aug 9;8(8):e012308. doi: 10.1136/bmjgh-2023-012308 (PMC10414093; doi:10.1136/bmjgh-2023-012308)
Supplement: Supplementary data [file bmjgh-2023-012308supp002.pdf]

TableS2: Mapping of details of medical device governance including regulation and oversight from included literature.

Author/ Year	Medical Device definition	Medical device classification system	Availability of legal framework	Pre-market phase	Placing-on-market phase	Post-market phase	Other details of governance/ oversight	Other details of governance/ oversight	Policy/guide lines for donated MDs	Actors involved	Regulatory and other governance challenges	Opportunities to improve governance/ regulation
DeMaria et al[34] (2018)	A medical device can be described as any means of improving or monitoring patient health that acts on the body in a non-metabolic fashion.	8 countries reviewed (Egypt, SA, Nigeria, Uganda, Ethiopia, Malawi, Tanzania and Kenya). Only Malawi had no information. All 7 countries used a 4 tier classification system modelled after the GHTF A-D or the EU I-III system. A or I representing the lowest hazard devices and D or III representing highest hazard devices.	All countries studied (except Malawi) have national laws/legislation in place relating to medical device regulation. Most countries had oriented regulatory processes in the legislation to EU directives mainly or FDA directives.	In most countries, the MoH or a National Regulatory Authority regulate local manufacture. Legislation sets documented technical standards or norms which provide specifications, guidelines or characteristics, including testing methods and	Countries may not have the capacity to test safety and performance of many devices. To market MDs within a country, certification is required. This includes a free sale certificate and CE mark or FDA approval (Egypt, Kenya, Nigeria, SA) and Product certificatio	No details	Coupled with a proliferation of MDS and varying regulatory processes, the differences in regulations between countries oblige manufacturers to prepare a different dossier for each country, which constitutes a lengthy and costly process, leading to a disincentive to medical device companies to sell in some countries. They are also a deterrent to innovation and the development of new products.	A core requirement for regulation is the evaluation of intrinsic risk and expected benefit of all medical devices. According to the intended use, length of time used, interaction with the human body and other technical characteristics, the device is considered more or less risky for the patient and therefore classified accordingly.	All 8 countries in Africa reported to have had a policy or guideline for donated MDS but no details provided.	NRAs, IMDRF, GHTF, FDA, EU ABEC-African Biomedical Engineers Consortium formed to improve capacity of biomed engineers to regulate health products including MDs.	Despite having legislation to guide regulatory processes, most countries had limited capacity to carry out regulation of medical devices due to lack of investment and knowledge and skills of personnel.	Harmonizing regulations: Regulatory authorities will benefit in terms of improved expertise, collaboration with other regulatory authorities and operational efficiency through sharing of information and recognition of established regulatory authority decisions. "Open source medical devices"- reduce cost of development while maintaining regulatory processes and safety levels similar to EU devices.

				acceptance criteria, for the design and manufacturing of medical devices.	n and pre-shipment verification of conformity certification based on relevant ISO standards (Uganda).							
Dacombe et al[35] (2019)	None provided. HIVST kits are classed as in vitro diagnostic (IVD), that is tests on specimens taken from the body, and thus are considered medical devices by the International Medical Device Regulation Forum.	Medical devices are classified according to the hazard the device presents based on its intended use and the expertise of the user and the impact of the result. Due to the potentially severe outcomes of an incorrect result and its use by lay persons, regulators	No details	Unclear. Surveys of regulation across Africa have identified IVD regulation as a neglected area. Development of regulation for IVDs in the 3 countries ranged from none in one country (Malawi) to the	HIVSTs must not only demonstrate the stability and accuracy required for device registration, but also take into account mechanisms for ensuring the kit performs optimally in the hands of intended	Many low- and middle-income countries and donors use WHO PQ as a pre-requisite or substitution for device registration. However, PQ does not cover all monitoring of device performan	In Africa, external quality assurance programmes are largely run by the tertiary referral/national HIV reference laboratories or national reference laboratories and act as a post-market surveillance system in the absence of or, where available, in collaboration with IVD regulators. However, the potential rolein	WHO pre-qualification was recognized across all countries, as an important mechanism for ensuring the quality of test kits from manufacturers. Little mention of the existence of any regional bodies, but participants from all sectors were aware of the benefits of a	No details	MoH policy makers, medicine regulatory authorities, Laboratory staff, central pharmacy stores, WHO/UN, donors, National reference labs, National Bureau of Standards	Across all three countries, knowledge and understanding of IVD regulation and HIVST was limited. It was unclear to respondents who had the mandate for regulation, with several actors reported as involved in regulation. Regulators in all	All countries recognise the need for improved coordination of IVD regulations. Respondents recognised weak coordination between ministries of health, regulators and national reference laboratories and more interaction and collaboration was needed. Technical working groups with a mandate to focus on HIVST were seen as a way of

	would likely consider HIVST kits as a Class D (highest risk) medical device and therefore subject to the greatest degree of regulation		drafting of guidelines for pre-market regulation in the other two countries.	users. In developed countries, this process takes years. To speed up this process and make the evaluation more focused on low- and middle-income countries, in 2016, the WHO released the technical specifications series for the pre-qualification (PQ) of HIVST kits.	ce undertaken once a device is on the market (post-market surveillance) though an adverse event reporting system is in place.	post-market surveillance of HIVST had not been recognized by most regulators.	shared approach and were open to the possibility.		countries expressed a need for more support to develop IVD regulations. None of the countries had regulations that entirely covered the regulation of IVDs or any specific guidance on HIVST regulation. A key concern was the potential entry of unregulated and risk of poor quality HIVST kits (particularly their performance in the hands of intended users) into the domestic market,	coordinating the development of policy and regulation while MOUs could serve as a way for different orgs to work together.

											mainly through the private sector.	
Dube-Mwezi et al[36] (2020)	No details	None specified.	7 countries within the region were reported to have a normative legal framework for medical devices	Reliance on the technical reports of the notified bodies in producing regulatory outcomes (in some cases with specialised multi-disciplinary expertise required to ensure appropriate oversight of their safety, quality and performance).	The regulation of medical devices mainly focused on registration and licencing (import and export) control, and to a lesser extent clinical testing and post marketing surveillance.	see previous column	Essential and complementary medicines constituted the greater part of the scope of product regulation for the majority of NMRAs compared to medical devices.	Efforts have been focused on strengthening regulatory frameworks for medicines, whilst focus on medical devices and in vitro diagnostics (IVDs) has not been equally high. Investing more efforts into strengthening frameworks for medical devices could be recommended as a priority for the SADC countries.	No details	NMRAs	Need to transition from the broad strengthening of the regulatory systems in terms of the scope and type of product, as well as specialisation of the regulatory functions. for example, other targeted forms of pharmacovigilance.	The core functions of NMRAs need to be expanded across the region, and in particular, to include product-specific expertise. It is also critical to bridge the gaps by making the mandate of the regulatory authorities comprehensive to encompass all types of medical products including medical devices.

Hubner et al[37] (2021)	Same as WHO definition	Most countries used a 4 tier risk based classification system in line with WHO or GHTF recommendations. South Africa was the only country that includes specific guidelines governing the regulation of in vitro diagnostic devices.	All COSECSA member countries, and South Africa, with the exception of Burundi,Malawi and Mozambique have legislation mandating the regulation of medical devices. SA's framework closely resembles IMDRF guidelines while other countries like Zimbabwe, Rwanda, South Sudan and Burundi use a more limited legal framework restricted to the mention/definition of	Conformity assessment requirements varied between countries. Countries like SA, Kenya, Tzn require device vendors to demonstrate conformity to WHO guidelines or to a quality management system used in IMDRF countries. Other countries like Zim focus assessment on devices such as gloves and condoms due largely	Many countries require devices, manufacturers, importers, and distributors to be registered with the national medicines regulatory authorities. Again Zim only requires this for vendors of condoms and gloves. In addition, over half of the countries had import controls in place to ensure MDs are approved before their	SA employs extensive post market controls including inspection per quality management systems procedures and guidelines, the seizure of devices that are unregistered or expired, reporting of adverse events, and controls of labeling and advertising. Other countries (Kenya, Ethiopia, Sudan, Tanzania, Uganda, and	Regulatory oversight for MDs in African countries remains limited and not well defined. Many countries look to international guidance such as from US FDA and EMA. These processes are stringent but costly and may discourage local manufacturers and developers. They may not be well suited for needs and issues of African settings. All countries except Mozambique (uses MoH) had a national medicines regulatory authority which regulated medical devices. The practical enforcement capacity of	Underdeveloped regulatory processes present challenges for businesses and manufacturers of new medical devices interested in entering the African market, as regulatory processes are country-dependent thus requires evaluating local laws and regulations on a country-by-country basis.	Implied as imported devices in the paper to which import controls will apply	NRAs	The majority of COSECSA member countries currently do not effectively regulate medical devices, due in part to both underdeveloped regulatory frameworks and a lack of downstream enforcement. Using FDA and EU approvals can be challenging as the review process may not consider infrastructural limitations currently present in many African nations. Many	Approaches to build state capability and subsequently expand the capacity of COSECSA member countries to regulate the marketing of medical devices include: an institutional approach which encourages the implementation of “best practices” from developed countries with a focus on improving regulatory capacity, but this is critiqued for not prioritizig country specific issues.

			medical devices, but does not assign specific responsibilities or guidelines for regulation.	to their role in preventing the transmission of HIV/ AIDS.	shipment and entry	Zambia) likewise have postmarket controls to varying degrees related to inspection but limited details available.	these bodies however remains limited.				medical devices designed to meet the standards of other countries have been observed to easily malfunction due to such factors.	
Kedwani et al[38] (2019)	None provided	Most countries use a 3- 4 tier based system. Risk is a combination of the probability of occurrence of any harm and severity of that harm. Two classification systems are used- either rule-based, or group-based. The former defines specific rules, which must be applied to	No details	Manufacturers require product registration and need to determine the national regulatory requirements and if market placement is possible. This is challenged by limited information sources. The diversity of	All manufacturers looking to place a product on a market should develop technical dossier and at least include the requirements of well-known harmonization groups such IMDRF.	No specific details	There is a gap between developed countries with stringent regulatory requirements for MDs and developing countries with limited to no regulations. Most national regulatory frameworks are concerned with protecting the population from harm. Generally, resources are focused on high risk products		No details	NRAs, manufacturers, harmonization working groups	Predominantly, the regulation of (MDs)and (IVDs) in Africa is weakly defined. Trained personnel or laboratory facilities for the correct handling of some diagnostic tests are often missing. Regulations are reinforced for MDs	

	identify the final risk classification. The latter defines product groups which are already assigned to a risk class. By the assignment to a product group the risk class can be determined.	the regulatory requirements across African countries complicates product registration.	with minimal control for low risk products, but these may come with some challenges to quality and safety.		used in specific infectious diseases such as tuberculosis, malaria or HIV/AIDS, due to the support of donor organizations

McNerny and Peeling[39] (2015)	No definition given but MDs broadly categorised: (1) active implantable (eg, cardiac pacemakers); (2) general medical devices (eg, scalpels and scanners); and (3) in vitro diagnostics (IVDs). The major distinction between the 3 categories is that IVDs use specimens taken from the body whereas the other	Most NRAs adopt a 3-4 tier risk classification system ranging from low, moderate and high risk, where risk is a combination of the severity of harm and the probability of its occurrence. Regulators need to decide whether the potential benefits of using a medical device or IVD outweigh the potential risks to both the individual (personal health) and to the public's health.	No details	Manufacturers seeking pre-market approval for a high risk diagnostic test would be required to submit a dossier compiling a full description of the product, the manufacturing process, a statement of intended use, copies of package inserts and user instructions, and evidence of analytical accuracy	Mainly advertising controls that prevent misleading claims about test performance and ensure clarity of intended use. African NRAs may take WHO prequalification, or approval by well-established NRAs such as the FDA and European Union (CE marking), into consideration when reviewing applications, thereby accelerating the	Post market vigilance ensures that satisfactory quality is maintained throughout the life of a product, either by active surveillance measures, such as batch testing to check quality, or by monitoring complaints. No details of whether this occurs in the region.	Other risks for diagnostic tests to consider in regulatory assessments is the risk fo incorrect or misleading test results.	In the absence of formal transparent mechanisms for regulatory control, some national disease control programs or Ministries of Health oblige companies to undertake product evaluation studies in a local laboratory, which can result in unnecessary and costly duplication. Tensions were evident between organizations whose primary function was to oversee the technical personnel in such laboratories	No details	NRAs, Pan African Harmonization working party (PAHWP). EU regulatory bodies and FDA.	The current regulatory landscape for diagnostic tests in developing countries acts as a disincentive to innovation and a barrier to new diagnostic products entering those markets. Although most African countries have national bodies that oversee the registration of medicines, few have capacity to regulate IVDs.	Mainly through harmonization efforts to shorten approval process time and save costs. Transregional efforts through the Pan African Harmonisation Working Party on medical devices and diagnostics (PAHWP)- a voluntary body that aims to improve access to safe and affordable medical devices and diagnostics in Africa through harmonized regulation. Priorities include establishing a laboratory network and communications platform for postmarketing surveillance activities and reducing unnecessary duplication in

	medical devices are used either in contact with or implanted into the body		and clinical performance. The NRA will also examine the quality management system of the manufacturer, including reports from a site visit by a team of experts. Unclear which countries in the region require this.	process of placing on market.		and regulatory agencies whose primary activity was the regulation of medicines.		clinical performance studies. However, harmonized approval are yet to be a reality.

McNerny et al[40] (2014)	Same as WHO definition	GHTF classification according to risk of causing harm. Stringency of regulatory oversight required is related to the harm that a false positive or false negative test result may cause to either individual or public health. High risk tests require more stringent control, including evidence of their performance as obtained through clinical studies.	National Regulatory Authorities (NRAs) are usually mandated by law. Most countries have a legal framework and a nominated body to regulate medicines, but regulation of medical devices in developing countries is less common.	Pre-market evaluation to assess safety, performance, benefits and risks prior to approval to market the device. Submission dossiers are unique to the country's regulatory framework and companies seeking approval to market an IVD are required to supply a dossier to the appropriate NRA describing the device and documenting	These stipulate conditions under which devices can be offered for sale; identify who may use the device and under what conditions; and to avoid inappropriate marketing or misleading claims regarding test effectiveness.	Currently, there is limited capacity for post-market surveillance in much of the developing world. Systems for post-market surveillance for diagnostic devices are not implemented, reporting and information sharing occurs on an ad hoc basis. In many countries, random quality checks, such as lot testing, are not	Current regulatory oversight of IVDs is variable and, in countries where there is no regulation, substandard and counterfeit tests may be sold openly. In countries that do regulate, approval for IVDs is often costly, lengthy and, on occasion, lacking in transparency, thus regulation of diagnostics is currently seen as a barrier to innovation and access	Current lack of standardisation across national regulatory authorities and the lack of clarity surrounding the regulatory pathways presents an unnecessary burden on manufacturers and acts as a deterrent to marketing in countries where financial returns may be modest.	No details	PAWHP members including national regulatory authorities, laboratory experts, manufacturers and international organisations	In most African countries, submission dossiers are unique to the country, with each NRA utilising its own indicators, nomenclature and format in its own language. NRAs in the developing world lack the expertise and capacity required in order to undertake audits of manufacturing facilities which ultimately results in delayed approval.	For IVDs used in developing countries there are opportunities to streamline and harmonise activities where convergence of protocols and mutual recognition of other regulatory bodies could improve their safety and quality, accelerating access to new tests while simultaneously minimising the costs incurred.

	evidence relating to the quality of manufacture, as well as the safety and stability of the components.	performed and tests can enter the market without checks on their quality. Most developing countries lack a feedback mechanism to provide manufacturers with information regarding the need for corrective action or mechanisms for withdrawal of substandard devices.	

Mori et al[41] (2011)	Medical devices (MDs) and in vitro diagnostics (IVDs): medical instruments, apparatus or materials used on patients for surgery, treatment, or diagnosis. Unlike medicines, their intended primary action is not metabolic, immunological, or pharmacological.	Not specified	No details	Large gaps exist in terms of quality assurance of MDs and IVDs. Most resource-limited countries lack the means to ensure appropriate regulatory control and are more exposed to the risk of low-quality products. No definition of poor-quality products have been agreed for MDs ⁄IVDs, making it more difficult to assess		Type of quality problems reported include the inappropriateness of sophisticated MDs ⁄IVDs imported and used in tropical conditions, poor technical performance, mislabelling including falsified expiry dates. Poor regulatory oversight and limited awareness of the problem among caregivers and decision makers.	Regulatory definitions of counterfeit IVDs- deliberately mislabelled products (e.g. by expiry date or product identity label), whereas substandard IVDs are those that (i) do not meet the specifications described in the literature or claimed by the manufacturer (e.g. as listed in the product information sheet) and ⁄or (ii) have apparent errors in labelling or on the product information sheet.	No information

				their presence and their impact on global health.								
Wong et al[42] (2014)	Medical devices are as defined by the WHO.	HIV RDTs have been classified as ''high risk devices'': results provided by these diagnostic devices have both serious individual and public health implications, and potential for harm due to false results (e.g. increased transmission).	No information	HIV self-diagnostics tests are legalised in Kenya and South Africa. n Kenya, HIV self-testing legalised since 2008 and included as part of national HIV testing policy. Should be subject to regulatory control but no specific guidelines are available.	Rapid HIV test kits are regulated within existing national policy frameworks, supported by WHO diagnostic pre-qualification and MOH validation of test kits. Currently no normative guidelines are available and no RDTs intended for self-testing	Tests are nationally approved only; vendor training and instruction are required. In Namibia and S/Africa, no specific guidelines, laws or policies for HIVST or quality of HIVST kits, although RDTs are regulated as medical devices. In all settings, private sector appears unregulate	Authors recommend HIVST regulatory oversight should include post-marketing surveillance to monitor the ongoing quality of tests sold, and laws that prevent low quality tests from entering the market. No specific guidelines available in the countries examined.	Increasingly, self- testing is also occurring through the private sector in resource-limited countries, where professional-use RDTs are varyingly packaged and sold directly to consumers. However, these services are often unregulated and not covered within national HIV policies, and the ability of governments to ensure appropriate, quality RDTs is limited.	No information	WHO, Ministries of Health and National disease control programs.	HIVST shows much promise, but regulations and policies still require adaptation to meet the specific challenges with RDT and self-tests. Regulatory frameworks supporting appropriate HIVST devices and self-testing implementation policies are both needed. Ensuring appropriate, safe, and effective diagnostics will require	Prioritise and strengthening regulatory infrastructures, longer-term strategies for strengthening country regulatory capacity for HIV diagnostics, including self-test RDTs. Others are policy development and decision-making related to the evaluation, sale or introduction of HIV rapid diagnostic tests intended for use by consumers. Regional regulatory-strengthening initiatives that support harmonised regulation of

	have been pre-qualified. Low resource countries considering HIVST will benefit from clear regulations and policies that ensure appropriate test kits are brought to market and quality will be ensured.	d, and it is legal for HIVST kits to be accessed through some stores and the internet but not in pharmacies.		Despite or because of this absence of regulation, RDT distribution continues through pharmacies, Internet sales, groceries and other businesses.		further development of current systems.	diagnostics using international and donor quality assurance processes that inform procurement decisions and harmonizing ‘‘minimum standards’’ of self-test devices.

Rugera et al[43] (2014)	Definitions for MDs and IVDs were same as the GHTF definitions.	In line with the GHTF A-D risk based classification system. The risk of causing harm is related to the performance of the device and likelihood of a malfunction and also the consequences of obtaining an incorrect test result for IVDs	Four Member States, Burundi, Kenya, Rwanda and Tanzania (Mainland and Zanzibar) reported legislation in the form of Acts of Parliament addressing products for health, including medical diagnostics and medical devices. Uganda did not have specific legislation for medical devices/diag nostics but legislation for drugs could be expanded to include other	With the exception of Tzn, premarket regulation of medical devices and IVDs by National Regulatory Authorities is largely absent across the EAC. No audit visits to manufactu ring sites are undertake n. Donated products supplied through vertical disease programs may receive some scrutiny, while others are approved	Most countries required manufactu rers to have a local agent with legal accreditati on prior to registering a product for distributio n in the country. Import authorizati ons were required in Tzn and Ugn with some enforceme nt by warehouse inspection s. Advertising controls were reported in Burundi, Tanzania and Uganda	Post market regulation was reported as being reactive rather than proactive i.e investigati ons were undertake n if problems were reported. The Private Health Laboratori es Board in Tanzania was the only organizatio n that reported a mechanis m for tracking medical devices or guidelines for	Limited capacity for regulation of medical devices or IVDs. Where medical devices are controlled it is largely within disease specific programmes such as tuberculosis, malaria or HIV/AIDS with the Department of Health or international donors guiding procurement decisions rather than guidance from a national regulatory authority. Government bodies will often have a list of approved products to guide procurement. Capacity refers to either lab based technical experience to regulate the use of MDs,	Some countries reported dual responsibility for diagnostic devices where more than one agency is mandated, and some tensions were evident between laboratory-based organizations who evaluate diagnostic test performance and agencies whose primary activities lie in the regulation of medicines. e.g policies are under review to better define the roles Kenya Medical Laboratory Technicians and Technologist Board	No information	NRAs of the 5 States, MoH, Kenya Medical Laborato ry Technicia ns and Technolo gist Board, Allied Health Professio nals Council Uganda	The need to strengthen existing National Regulatory Authorities was also highlighted. Suggestions for future harmonizati on activities included use of a common nomenclatur e and definitions, mutual recognition between EAC countries and reducing the number of clinical trials required for registration. Areas to improve training and capacity included dossier evaluation	Mainly through harmonization efforts. Priority to be given to rapid diagnostics for important infectious diseases (HIV, TB, Malaria) across Partner states with extension to other MDs in the longer term. To this end, all members need to have a policy and legal framework for regulation. Also establishment of a communications platform to share information about safety of MDs promptly. Donor funding to enhance capacity and training of NRA recognising expertise in clinical trials or review of submission dossiers.

	medicinal products.	without further testing. In few cases, local labs evaluate products. Products with international regulatory approval (Canada, Japan, Aus, USFDA or WHO pre-qual) can be approved using an abridged process.	with some vetting and pre approval required.	recalling substandard medical devices or IVDs. Most states had some mechanism in place for pharmaceuticals but not medical devices.	capacity to do lab assessment of IVDs or involvement in clinical trials to assess performance of MDS. Some capacity for clinical trials were reported for MDs used in vertical dx programs. Kenya and Uganda reported having accredited labs with Tzn working towards it.	(KMLTTB) which has responsibility for the quality of medical laboratory activities and the Pharmacy and Poisons Board which regulates medicines.		and review, development of protocols and Standard Operating Procedures, quality management systems and post market monitoring and surveillance.	

WHO[44] (2020)	WHO definition of MDs PMS includes all activities by the manufacturer to ensure that medical devices continue to be safe and well performing, and actions are undertaken if the risk of continued use outweighs the benefits. Receiving and evaluating feedback are the minimum requirements of the PMS	All medical devices, including IVDs, are covered by this guidance, without prejudice to national or regional legislation. However, national or regional legislation can require the manufacturer to perform more elaborate post-market surveillance	No information.	Feedback from manufacturers: perform monitoring by collecting and analysing experiences from actual use of medical devices. PMS may be reactive through passive collection and evaluation of feedback or proactive. PMS is linked to the overall risk management process of the manufacturer. Mnaufactu	NRAs raise awareness among users, should develop a system to receive feedback directly and ensure feedback Is forwarded to manufacturers/vendors. NRAs may conduct a risk assessment when forwarding feedback to ensure MD is registered/ authorized or regulatory action is required for unregistered/non-compliant	Although users have no official responsibility for post-market surveillance, most of the information on the experience with the actual use of medical devices will come from users. Users include; lay users/care givers, patients/clients including self-testers. Users detect/observe issues and provide feedback	What manufacturers/ vendors report to NRAs will depend on national legislation. Overall patient/user harm is considered. In general, incidents that involved a serious public health threat, result in death of a user, patient/client or other person; serious deterioration or indirect harm, such as misdiagnosis, delayed diagnosis, delayed or inappropriate treatment, etc. The timing of reporting will also depend on severity of the threat. Manufacturers	Receiving and acting upon user or other feedback is the most basic form of post-market surveillance that must always be performed by the manufacturer, irrespective of their resources. This means that the methods to submit feedback shall be readily available and provide as few barriers as possible to users and patients/ clients to provide the feedback.	No information	Manufacturers of medical devices, and their economic operators in the medical device supply chain; health care providers and their patients/ clients as users of medical devices; programme implementers, including procurement agencies and central medical stores; and NRAs.

	system. MS or vigilance refers to all activities of NRAs in the oversight of medical devices on the market, to ensure that the safety, quality and performance continues to be adequate.			rers collect and classify feedback and determine reportability to NRA. Next they determine if corrective actions are needed and implement these/other preventive actions.	devices. Due to limited resources (financial and human) a MS plan will be prioritized using a risk-based approach for closer surveillance including testing of MDs.	to manufacturers and NRA if possible. Manufacturers may provide advice on further actions to take. Registries are being increasingly used, especially for implantable medical devices, to collect data on clinical use and to assess use in the medical device's target population.	should inform affected users of any corrective actions taken in the field.

World Health Organization[45] (2003)	The Global Harmonization Task Force harmonized definition: "Medical device" means any instrument, apparatus, implement, machine, appliance, implant, in vitro reagent or calibrator, software, material or other similar or related article, intended by the manufacturer to be used, alone or in combination, for human beings for	Classification is based on risk assessment to categorize medical devices according to their perceived potential hazards. Potential areas of hazard considered include: the degree of invasiveness, duration of contact, body system affected and local vs systemic effects. The GHTF is proposing a harmonized classification system based on similar systems in the EU, US and Canada.	not applicable	Manufacturer to control/monitor product. Activities regulated include device attributes (safety, performance), quality systems and labelling to ensure accurate representation. All systems use the risk management philosophy with the degree of regulatory scrutiny increasing with the potential risks of the medical device.	Vendor establishment control and registration including listing of products available or in use, fulfilment of after-sale obligations (post-marketing surveillance) and appropriate advertising. Vendor information facilitates governments in tracking medical device vendors. A priority for local regulatory oversight	Surveillance/vigilance. Vendors have after sale obligations - monitor device clinical performance, identify problems and alert. Some countries have mandatory requirements for vendors or manufacturers to report all device-related events that have resulted, or could result, in serious injury or death. Developing countries should	Quality management standards for medical devices are issued by the International Organization of Standards. . The applicable standard is determined by the risk class of the device and depends upon the regulatory system of the country or region. Most countries will use IOS standards.	Government role in ensuring medical device safety and performance is through the implementation of regulation. 3 stages of regulatory control are described which correspond across the life span of a medical device. These are the pre-market and post market controls, and the placing-on-market controls, the last which is not an official term but related to the important aspect of how the product is represented	No information	WHO, Manufacturers, Vendors; Users including healthcare providers; Public which include direct users, National government.	Problems have risen with regard refurbished and donated MDs and equipment- often traded or donated to developing countries with questions around the quality, availability of after-sale technical support or spare parts. Not much work done for refurbished devices but WHO has worked to set out guidelines for dealing with donated devices and equipment. Currently,	Efforts by the GHTF and trends towards use of international standards and the recognition of international certification, creates opportunities for countries to establish low-cost programmes that promote the safety and performance of medical devices by taking full advantage of what others have already done in this field. Local adoption of harmonized recommendations will facilitate international exports of medical devices manufactured locally.

	one or more specific purposes and which does not achieve its primary intended action in or on the human body by pharmacological, immunological or metabolic means, but which may be assisted in its function by such means.		Authorities acknowledge product clearance for the market in various ways. eg EU CE Mark. Governments unable to carry out pre-market review, either for imported devices or those manufactured locally, could assure regulatory compliance by taking advantage of the work of major device manufacturing countries.	should be the establishment of vendor and product registrations.	prioritise user training and post-market surveillance of devices (correct use, problem alerts and recalls). International sharing of information on alert systems for medical devices is essential for more effective risk management.		to the user. Pre-market controls contributes to controlling the product to ensure safety and performance at the manufacturer stage. Post market controls ensure continued safety and performance in use.		WHO export certificates only apply to pharmaceutical products. Regulatory authorities may not have the resources available to provide details specifications on devices for regulation.	

WHO[46] (2017)	Same as WHO definition (2003). However, not all products will fit into this definition of medical devices comfortably. These are referred to borderline products for which it is unclear which legislation applies. A combination product is a product comprising two or more components which are regulated as medical	Generally, the risk of a medical device is determined by the potential of the device to cause harm to the patient/user. For IVDs, risk class is primarily determined by the impact of an incorrect result either on the health of an individual or the public. Classification system guides the regulatory controls to be implemented. Classes are broadly from A to D: A, low risk B low-med risk, C med-high risk and D high risk. Regulatory control	Medical device regulation must have a sound basis in law. The document recommends a phased implementation of regulatory control systems. The basic level is the publication of a law establishing a NRA and providing resources to it. Expanded levels includes inspection of regulatory establishments and oversight of clinical investigations. More broadly, regulation of medical	Manufacturers demonstrate compliance with GHTF guidance in the technical documentation shown to NRAs before or after introduction of medical devices to market. Some NRAs may appoint an external body for conformity assessment to assist in this function of pre-market approval. The manufactu	These include registration of establishments, listing of devices and import controls. The minimum requirements for registration should be that the authorized representative provides the regulatory authority with information on its place of business, the name and position of a responsible person and the	At the basic level the regulatory authority should establish a system whereby users, patients and the manufacturer of medical devices, either directly or through the authorized representative, can report complaints involving medical devices, including malfunction at the device level and adverse events at the patient	It is recommended that regardless of class, manufacturers should be ready to submit technical documentation where it is required to assure conformity to regulatory requirements.	International harmonization guidance documents (by the GHTF) have been developed for almost all basic and expanded pre-market controls. Controls for medical devices may be implemented through reliance or recognition. In addition to relying upon the work of other authorities, for some medical devices (mostly IVDs), the regulatory authority may choose to rely upon evaluations conducted by the WHO PQT	To safeguard public health, medical devices imported as donations should comply with all regulatory requirements on safety, quality and performance and should not differ from those that are imported through a regular supply chain.	NRAs

	products, i.e. medicine/ medical device, or vaccine/medical device. A lack of clarity in such cases may lead to overlapping or conflicting regulatory requirements for a product, or no regulation at all.	increases with class of device. The manufacturer has the primary responsibility to classify the MD but the decision may be challenged by the local NRA.	devices should be coordinated with that of other medical products e.g medicines and vaccines, and with wider government policy objectives.	rer demonstrates conformity through its quality management system. Class A devices require no submission of dossiers or documents for pre-market approval except where sterility or accuracy of a measuring function is required. Class B devices do not normally require pre-market review but this may be	manufacturer it represents	level, in particular those adverse events resulting in death or serious injury. Vigilance reports may trigger investigation, trend analysis and/or possible field safety corrective actions or enforcement.		for IVDs. The focus of this programme is on IVDs for priority diseases such as HIV/AIDS, malaria, hepatitis C and others, and their suitability for use in resource-limited settings

	requested to verify compliance. Class C and D devices require an in depth review of technical documentation prior to approval and either the authority has confidence the QMS system is appropriate or conducts an audit before market authorization is given.	

WHO[47] (2016)	A medical device is any device or machine, tool or application for medical use, whether alone or with any other supplements such as those required for special applications are running, which are developed for human use.	Group I, IIA, IIB, and III	Medical Device Regulations (2010)	Yes - Depending on the risk class of the device, the conformity assessment procedure varies. (E.g., A Class I non-sterile, non-measuring device requires a declaration of conformity before it may be placed on the market.). Egypt relies on the highest health authority in US and EU jurisdictions to issue a certificate	The manufacturer must collect data in one of two situations: (1) as a condition of product approval, and (2) to re-affirm product safety when post-market adverse incident reports suggest that pre-market safety claims are inconsistent with actual use and result in unacceptable risk. Guideline for Medical Device	Adverse event reporting: The manufacturer must: - have suitable vigilance systems in place, - Notify the Medical Device Safety Department (MDSD), - investigate and assess incidents, - submit a trend report to the MDSD when reporting criteria are met as well as a periodic summary report. Users are also encouraged to report suspected incidents to the manufacturers.	Guidelines on a Medical Device Vigilance System, Art. pg. 27. Field safety corrective action monitoring: Manufacturers must notify the MDSD of any Field safety corrective actions (FSCA) of their products, take all necessary corrective actions, issue a field safety notice, and distribute that notice to organizations and users. Guidelines on a Medical Device Vigilance System, pg. 7	No information	Medical Device Department, Egyptian Drug Authority. The Medical Device Safety Department (MDSD), within the Central Administration of Pharmaceutical Affairs (CAPA), is a separate entity slated with monitoring the medical device market in Egypt.

		of free circulation. This Free Sale Certificate is necessary for any product entering into the market.	Vigilance System, pg. 5. The departmen t of medical device inspection conducts the inspection on local manufactu rers aiming to ensure that they apply GMP regulations : Initial Periodic (routine inspection) . In addition, the departmen t inspects stores of imported medical devices.			

WHO[48] (2016)	Similar to FDA definition: A medical device refers to an instrument , apparatus, implement , medical equipment , machine, contrivanc e, implant, in vitro reagent, or other similar or related article, including any componen t, part or accessory, that is: a) recognized in a pharmaco poeia or any supplemen t to it; b) intended	Class I, II, III, and IV. Classification depends on the indications for use, duration of use, degree of invasiveness, and local vs. systemic effect of the device. Classification rules are detailed in Annex II of Guideline (Sept. 2014)	A Proclamatio n to Provide for Food, Medicine and Health Care Administrati on and Control, Proclamatio n No. 661/2009 with Guidelines for Registration of Medical Devices.	Medical device essential safety and performan ce requireme nts are listed for MDs and IVDs but no other details of pre-market evaluation given	No import controls. Registratio n of establishm ent: An agency agreement should be made between the manufactu rer of the medical device for registratio n and the agent responsibl e for the import, distributio n, and sale of the product in Ethiopia. Guideline (Sept. 2014), Section I, Art. 2 Listing of medical devices:All medical	Prior to and after placing the product on the market, the manufactu rer should put a process in place, as part of its quality manageme nt system, to assess the continued conformity of the device to the essential principles of safety and performan ce through the post-marketing phase. Guideline (Sept. 2014),	Inspection (QMS): The manufacturer should always provide certification of conformity against internationally recognized standards for all class devices. The adequacy of the standards in relation to safety and performance of the device should be discussed with relevant supporting data for Class II and higher devices. In case the provided certification is found to be unsatisfactory, the Authority may conduct an onsite audit and inspection of the facilities of Class III and IV device	Adverse event reporting: Both the manufacturer and the NRA must sign an agreement that they are both responsible for post-marketing reporting of the device. Guideline (Sept. 2014), p. 26	No	NRA

	for use in the diagnosis of disease or other conditions, or in the cure, mitigation, treatment, or prevention of disease, in man or other animals,, or; c) intended to affect the structure or any function of the body of a human being or other animal and which does not achieve any of its principal intended purposes		devices should be registered with FMHACA. Guideline (Sept. 2014). Labelling: Labels may appear on the device, on packaging, or as instructions for use. They must: - be in English and/or Amharic - not be presented in a false, misleading, or deceptive way - be appropriately formatted etc. Guidelines (Sept.	Section II, Art. 2.2	manufacturers. Unless it is deemed to be necessary, the QMS of Class I medical device manufacturers' facilities are normally not subjected to onsite inspection. Guideline (Sept. 2014), p. 40, 50.	

through chemical action within the body of the human being or other animals and is not dependents upon being metabolized for the achievement of any of its principal intended purposes.	2014), p. 34-38

WHO[49] (2016)	similar to FDA definition above	Class I, II, III, and IV.	Food and Drugs Act (amended 1996), with Guidelines for Importation and Registration of Medical devices. This establishes the Food and Drug Authority with the Medical Device Department responsible for regulation and oversight of medical devices.	A manufacturer must submit an application prior to import, which includes a declaration of conformity to the Guideline for Registration of Medical Device. Medical devices can be registered provided that they comply with the prescribed standards and that the manufacturing operations for the article	Import controls: All imported medical devices must be registered and accompanied by a certificate of analysis issued by the competent regulatory authority of the exporting country. The Food and Drugs Authority issues a license for importers. Advertising: Deceptive advertising of a medical device is prohibited. One may not	The relevant body must appoint a local representative, who monitors the safety of a product after marketing approval. Adverse event reporting: An appointed local representative must report adverse effects or events to the Food and Drugs Authority. The Authority will monitor the safety of the medical	Registration of establishment: A person may not manufacture for sale, sell, supply or store medical devices in premises unless registered. Listing of medical devices: The Food and Drugs Authority must register the medical device if the Authority is satisfied that the medical device complies with standards. All devices that are manufactured, prepared, imported, exported, distributed, sold, supplied, or exhibited for sale must be registered with the Authority.		No information	NRA: Medical Devices Department (MDD), Food and Drugs Authority Ghana	

	comply with the prescribed current code of good manufacturing practice. One may not conduct a clinical trial of a medical device without an approved, valid certificate issued by the Food and Drugs Authority. No reliance on other approvals were directly mentioned.	advertise a medical device without the prior approval of the Food and Drugs Authority. Deceptive labelling of a medical device is prohibited. The label of the medical device must be in English and include information on the name of the device, the name and address of the manufacturer, the identifier of the device, the	devices through analysis or report and take appropriate action when necessary. Field safety corrective action monitoring: The Food and Drugs Authority may order the closure of any premises where medical devices are manufactured, stored, prepared or sold if the Authority has reason to believe that the articles are at risk of

					batch number (for class III or IV) devices, etc.	contamination or deterioration.					
WHO[50] (2016)	same as WHO definition	not available	Yes and NRA is available.	Medical devices may not be imported, placed on the market, or put into use unless there is a certificate that shows their compliance with essential requirements on the health of		The failure to report adverse events may result in a fine and imprisonment based on Public Health Law, Art. 382. Manufacturers, users, and those familiar with any adverse event must			No	NRA	

	patients. The ministry of health provides the certificate. The manufacturer is responsible for meeting conformity requirements, as set by the Ministry of Health Public Health Law, Art. 379.	report that event to the Ministry of Health. Public Health Law, Art. 381.	

WHO[51] (2016)	A medical device means any instrument, apparatus or contrivance (including components, parts and accessories thereof) manufactured, sold or advertised for internal or external use in the diagnosis, treatment, mitigation or prevention of any disease, disorder, abnormal physical state or the symptom thereof, in man or	no detail	National Agency for Food and Drug Administration and Control Act Cap N1 Laws (2004), with Guidelines for the registration of imported medical devices in Nigeria.	All medical devices must be registered to be manufactured, imported, exported, advertised, sold or distributed in Nigeria. An application for registration must be made either by the Nigerian manufacturer or through an authorized representative for a manufacturer outside of Nigeria: Guidelines for Registration of Imported	Import controls: The Ports Inspection Directorate inspects and controls the importation of medical devices. Advertising :The Registration and Regulatory Affairs Directorate implements advertising controls of regulated products, which include medical devices. Labelling: All labels must be clear and informativ	The Pharmacovigilance/Post Marketing Survey (PV-PMS) Directorate provides post marketing surveillance of NAFDAC regulated products, which include medical devices, and maintains a database on adverse events.	no

animal. CAP N1 Laws, Art. 31		Medical Devices in Nigeria.	e and include at minimum: - name of the product - name and address of the manufacturer - NAFDAC registration number - batch number, manufacturing date, and expiry date - net contents - directions for safe use. Any regulated product in a foreign language will not be considered for registration unless an English translation is included.

WHO[52] (2016)	none available	not available	Medicines and Allied Control Act, Chapter 15. Of Medical devices is focused on only two types of devices, condoms and gloves, which are regulated in Zimbabwe with separate guidelines. Medical devices unit carries out quality control testing of condoms and gloves in line with international standards. Physical tests are carried out on the medical devices as well as	No person shall sell any condoms/gloves unless such is of a type and brand which has been approved by the Authority. A register of approved condoms and gloves.	Import controls: Port Authorities shall not approve the importation of gloves batches unless the NRA has approved such importation. No guidelines for advertisement and labelling.	Devices may be subject to inspection. Any person who resists, hinders or obstructs an inspector, customs officer or police officer in the exercise of his functions under this Act shall be guilty of an offence and liable to a fine or to imprisonment under Medicines and Allied Substances Control Act, Art. 67. Adverse			no	NRA: Medical Devices Unit, Medicines Control Authority of Zimbabwe

	checks for poor workmanship.	event related to medical devices can be reported to the MCAZ on its website. Where the Authority is of the opinion that the withdrawal of any batch of condoms or gloves is necessary for the protection of the public, the Authority may require any person to withdraw such batch in accordance with the procedure	

						as determined by the Authority. See Medicines and Allied Substances Control (Condom) Regulations, 2005 and Medicines and Allied Substances Control (Gloves) Regulations, 2006.			
WHO[53] (2016)	None provided	not available	There is no legal framework. The Pharmacy Management, Equipment, Supplies and Medical Supplies division of the MOH is charged with overseeing	No details	No details	No details		No	MoH

			quality control of medical devices imported and locally manufactured					
WHO[54] (2016)	A medical device means any instrument, appliance, material, machine, apparatus, implant or diagnostic reagent used or purported to be suitable for use for medical or veterinary purposes, and includes a part or an accessory of a medical device.	Unclear, there is a Medicines and Related Substances control Act 2003 but this does not include medical devices. Namibia Medicines Regulatory Council has no specific responsibilities assigned in regulation of medical devices.	The Namibia Medicines Regulatory Council purports to register medical devices, but there does not appear to be any legislative mandate or form to apply to register.	No details	No details	No details	No	NRA

	Medicines Act, Art. 1.							
WHO[55] (2016)	same as WHO definition	not available	No details but a decree/law established the NRA which is the Agency of Regulation and Supervision of Pharmaceutical Products and Food.	No details	No details	No details	No	NRA

WHO[56] (2016)	Similar to FDA definition	Class A, B, C, and D. Classification is based on the GHTF rules.	Tanzania Food, Drug, and Cosmetics Act FDCA (2003). There are guidelines on Submission of Documentation for Registration of Medical Devices (Oct. 2009); Tanzania, Food, Drugs and Cosmetics (Control of Medical Devices) Regulations, 2015. The NRA is Tanzania Food and Drug Authority.	A person must have an appropriate license to manufacture for sale, sell, offer, supply, or import a medical device. FDCA, Art. 22. Exceptions are made for custom-made devices. One must provide a declaration of conformity that contains an attestation that a device complies with applicable internation	Registration of establishment: A person may not sell, supply or store a medical device except in registered premises. Listing of medical devices: If the TFDA approves registration, then it will enter in data on the medical device into the register, assign a registration number, and issue a certificate of registration. The TFDA	PMS and Inspection of facilities for quality management systems is conducted. To enforce, there are various provisions that impose financial penalties on violations of the FDCA such as fines and imprisonment in some cases. The Minister, after consulting with the Director General, may make regulations to prohibit,	False advertising is prohibited. One must obtain written approval for promotional activities. Labelling: No person may sell any registered medical device unless it is labelled with the registered name, number, and directions for use in English and/or Kiswahili. see also Guidelines, p. 24. No person may sell or supply a medical device that is marked or labelled in such a way that it falsely describes the product or is likely to mislead.	Clinical investigation controls: To conduct a clinical trial, one must be (1) the holder of a product registration that authorizes a clinical trial and (2) also the recipient of a "Clinical Trial Certificate." Further, one may only conduct a trial of a medical device with the authorization of the Director General. To apply to conduct a clinical trial, a person must submit an application that includes an Ethical Clearance Certificate.	No details	NRA: Tanzania Food and Drug Authority .

	al standards. Tanzania relies on conformity assessments of other countries, but jurisdictions were not specified.	registers all medical devices it approves for use in clinical trials. Some low risk medical devices (i.s. class A) need not be registered. Guidelines, p. 12. Exemption from registration does not also discharge legal obligations of medical device dealers to keep records, report adverse events, and recall devices. Import	control, or restrict manufacture, dispending, possession, sale or use of a medical device. Further, if the Minister finds that a medical device lack claimed therapeutic value, he or she may prohibit the manufacture, sale, or distribution of the device. An inspector, upon finding a product is unfit or does not meet requireme		Once the TFDA receives the application, it will conduct an investigation to authenticate the safety, efficacy, and quality of the medical device and then register the product for purposes of clinical trials. The TFDA monitors all stages of the clinical trial to ensure protection from adverse events.	

	controls: A person must have a license to import a medical devices	nts, may - affix a mark to the device - destroy the product. An inspector may take any sample for analysis. He or she may also enter the premises entered on the register or on the license and examine any certificate, license, or other informatio n. The inspector may also close premises found to contraven e the law.	

WHO[57] (2016)	same as WHO definition	Class A, B, C, and D. There was indication of classification rules in the guidelines	The Pharmacy and Poisons Act, Chapter 244 (2002). There are Guidelines on Submission of Documentation for Registration of Medical Devices (2011). The NRA is Kenya Pharmacy and Poisons Board which supervises medical device regulation	Medical devices must meet essential principles of safety and performance. No conformity assessment details given nor requirements for registration and listing. It was stated when the medical device proves compliance to applicable essential principles and gets approval of the committee it will be granted a registratio	In terms of imports, One may freely import a medical device after being granted a registration certificate and complying with post marketing requirements.	Manufacturers and local authorized representatives must meet post-market requirements that consist of distribution record-keeping, records of complaints, adverse event reporting. PPB may send an QMS inspection /Auditing group to Class C and D manufacturers abroad to check their quality assurance system based on Kenyan	Manufacturers and local authorized representatives must maintain records of adverse events and to notify the PPB of any adverse events related to the failure of a device or a deterioration of its effectiveness, etc Users have the primary responsibility to report to PPB and the manufacturer of any adverse event. The manufacturers and local authorized representatives must have field safety corrective actions in place.		No	NRA: Poisons and Pharmacy Board

	n certificate.	national standards and other relevant medical device standards and registered product standards. The failure to report adverse events may result in fines, stopping the affected product, field safety corrective action (FSCA), and/or temporary withdrawal or loss of an operating license.	

WHO[58] (2016)	Definition of a medical device is harmonized with the GHTF definition. IVDs are defined separately and harmonized to EU Directive 98/79/EC.	Burkina Faso has a risk-based classification system Class A,B,C,D. Classification rules or other details not available	The Joint Order No.537 2013 on the regulation of in vitro diagnostic medical devices (IVDD) and medical consumables and Joint Order No. 2013- 1125 / MS / MEF on the conditions for the granting, withdrawal and renewal of technical approval for the supply of reagents and medical consumables, and the supply, installation, commissioning and maintenance of material and medical-	There is a conformity assessment procedure for IVDs with reliance on EU/US FDA assessments. Clinical investigation control are in place. Registration and Listing is required for some medical devices. Criteria are based on the risk classification of medical devices	Import controls: Each importer must submit an importation application to verify that the IVD is registered. All other medical devices are merely listed when imported. No regulations for advertising but guidelines exist for labeling of medical devices for sale in the market	Adverse event reporting: Burkina Faso has adopted the EU definition of an adverse event. Manufacturers, importers, wholesalers, distributors, and users are responsible to report adverse events. Field safety corrective action monitoring: Manufacturers, importers, wholesales, distributors and end-			No	NRA: General Directorate of Pharmacy, Medicines and Laboratories

			technical equipment.			users are mandatorily responsible for carrying out Field safety corrective action (FSCA) for medical devices.		
WHO[59] (2016)	Any equipment, device, instrument or product, with the exception of human origin products or other article used alone or in combination, including accessories or software interfering in its functionin	Non stated	Order of Aouel Dhou El Kaada 1429 (2008).	Medical devices require approval before being placed on the market, and there are import controls in place	Advertising of products must be submitted in advance to the agency. Labelling: Medical and scientific information on medical devices is mandatory. It must be accurate, auditable and compliant	No details	No	NRA: Department of Pharmacy and Medicines, Ministry of Health, Population and Reform

	g for use in humans for purposes: -diagnosis, prevention, monitoring, treatment or alleviation of disease or compensation for an injury or handicap, -study, replacement or modification the anatomy or of a physiological process, -control of medically assisted procreation		with the most recent medical and scientific data.

Peeling Rosana[60] (2015)	None provided	GHTF classification which considers both personal and public health risk. Class A termed low, Class B moderate, Class C Moderate-High and Class D- High.	Not information	The Pan African Harmonization Working Party (PAHWP) was set up in 2012 under the African Union-New Partnership for Africa's Development (AU-NEPAD) agency. It leads and cooridinates regulatory harmonization activities for medicines, medical devices and diagnostics in the African region, in	PAHWP focuses on several priority areas across countries including: a common risk classification system through the adoption of the GHTF system; common dossier template for device registration through the adoption of the WHO-PQ dossier for IVDs/diagnostics; QA system audits through convergence on	Post marketing surveillance through regional lab networks to monitor test quality and assurance of quality of diagnostics is recommended.		PAHWP focuses on several priority areas across countries including: a common risk classification system through the adoption of the GHTF system; common dossier template for device registration through the adoption of the WHO-PQ dossier for IVDs/diagnostics; QA system audits through convergence on inspections to manufacturing sites using preapproved standards/protocols set by IRBs and joint review of data		PAWHP member countries and regional groups, WHO, international organisations	Top 5 challenges for IVD regulations include: regulatory landscape for IVDs highly variable; assessment of safety and quality based on risk classification but often lack rigour; the process of approval is not transparent; Approval is often costly and lengthy, especially for imported tests; limited success with standardisation and harmonization	Strengthening regulatory oversight in Africa through harmonization approaches is key. Supporting the PHWP, AU-NEPAD and regional economic communities is important. Similar initiatives undertaken by 4 SA countries (Zambia, Zimbabwe, Botswana and Namibia) to collaborate and share information on medicines regulation through their NRAs called the Zazibona initiative. Harmonization will result in streamlined regulatory processes, improve access and affordability of MDs, enable

	partnership with the WHO and other international groups. 23 countries are members including the East African Community and SADC, along with the African Society for Laboratory Medicine (ASLM), GIZ, LSHTM. The EAC acts as chair with Nigeria and SA as Vice Chair and Secretary respectively.	inspections to manufacturing sites using preapproved standards/ protocols set by IRBs and joint review of data from site evaluations, as well as convergence on standards and recognition of third party audits; reduce duplications in clinical performance studies and trials		from site evaluations, as well as convergence on standards and recognition of third party audits; reduce duplications in clinical performance studies and trials; post marketing surveillance through regional lab networks to monitor test quality and assurance of quality of diagnostics.		faster approvals and access to quality assured devices, save companies time and money, lead to better patient outcomes and support innovation.

WHO[61] (2016)	A medical device means any instrument, appliance, material, machine, apparatus, implant or diagnostic reagent…or declared by the Minister by notice in the Gazette to be a medical device and includes any part or an accessory of a medical device.	Class A, B, C, and D General Regulations, Art. 12. with classification rules specified.	Medicines and Related Substances Act of 1965, Act No.101. with General Regulations Relating to Medical Devices and In Vitro Diagnostic Medical Devices [hereafter General Regulations]	Use of Notified bodies for conformity assessments must be approved by South Africa. One must apply to the Council to conduct a clinical investigation on an unregistered medical device or on a new intended purpose of medical device or IVD.	Manufacturers must obtain a license, import and/or export a medical device and/or IVD in South Africa. All medical devices, except custom made devices, and all IVDs shall be registered with the Council. Permitted advertisements to certain audience (i.e. public vs. health professionals) vary according to the classificati	Inspection (QMS): As part of an application to register a medical device, the manufacturer must certify a QMS is in place. Premises are subject to inspection. Enforcement: Medical devices or IVDs may be seized if they are unregistered and sold in contravention of the Act, suspected counterfeit, expired, etc. General Regulations, Art. 16.				No information	NRA: Medicines Control Council (MCC)

					on of the medical device or IVD. Nonetheless, no advertisement may be false or misleading. The label of a medical device should be in English.	One who fails to comply with the Act may face a fine and/or imprisonment. The applicant or holder of a registration certificate for a medical device or an IVD must inform the Council of suspected adverse events that result from that device.				
WHO[62] (2016)	none	not available	Unclear. The NRA is responsible for the registration of drugs and other health products as	No details	No details	No details			No	NRA

			defined in the Health Code in order to grant them a Certificate for Marketing (AMM) in Madagascar								
WHO[63] (2016)	Medical device means any instrument, apparatus including components, parts and accessories of it, or medical consumables manufactured, sold or represented for use in the diagnosis, treatment, mitigation or prevention of a	not available	The Pharmacy and Drugs Act, No 58 2001 includes some guidelines for medical devices. These are guidelines for conducting clinical trials of medical devices, registration of medical devices, and licensing of manufacturing industries in Sierra Leone. The NRA is	Manufacturers must apply for a license to manufacture a product based on guidelines for Licensing of Manufacturing Industries. Manufacturers are required to have and report on their quality management system.		There is a guide for Detecting and Reporting Adverse Drug Reactions and Field safety corrective action monitoring. Manufacturers shall have arrangements and recording system for handling of complaints, distribution of			No	NRA	

	disease, disorder or abnormal physical state, or the symptoms of it in man or animal. A medical device can be: (a) Condom (b) Glove (Surgical/ Examination) (c) Test - Kit (d) Needle and syringe (e) Insecticide Treated Net (ITN) etc.		Pharmacy Board of Sierra Leone, Ministry of Health and Sanitation.			products and product recall, when necessary.				
WHO[64] (2016)	same as WHO definition	Yes Categories: Class I, IIA, IIB, and III. Medical devices are classified by function according to: - the duration	Law n ° 84-12 relating to medical devices. NRA is the National Advisory Commission on Medical Devices. It	Whoever manufactures, imports, exports, or distributes medical devices must submit a	No import controls. Listing of medical devices: One must receive a certificate of registratio	Post Market Surveillance: Law n° 84-12 institutes a national system of market surveillanc	Medical devices must meet high safety level of use for the patient, professional and meet the essential requirements of quality, safety		No	NRA

	of use; invasiveness; the means of use (surgical or not) and use on the body.	gives opinions on Applications for the registration of medical devices; The suspension or revocation of registration; Withdrawal of a medical device from the market for public health reasons; Advertising visa applications and decisions to revoke those visas.	declaration of that activity. Registration of establishment: Not applicable.	n before one may place a medical device on the market. Certain medical devices are exempt from the registration requirement, including device intended for experiments/research and custom-made ones. Advertisements for medical devices may not be deceptive and must show good usage of	e. Inspectors are authorized to conduct periodic inspections of the manufacture, import, export, distribution and maintenance of medical devices. The administration, after consulting with the national commission on medical devices, may order those devices off the market. Fines are available for violations	and performance set by regulation.

					the device. Each medical device must be accompanied by instructions or label with sufficient information for safe use and to identify the manufacturer.	of certain provisions of the Law. One who manufactures, imports, exports, or distributes medical devices as well as professional users of medical devices must report within 48 hours of learning of any adverse incident or risk of incident when using the device.					
WHO[65] (2016)	Referred to as Medical Supplies: supplied products to be used	Categories: according to GHTF and FDA principles. Law on Registration of Medical	Law on Registration of Medical supplies 2010. Essential principle is	Outside Sudan, the medical supply should be registerd in the	Manufacturers should be registered in the country with listing	Field safety corrective action monitoring: When products			No	NRA	

	in medical environments.	supplies 2010, Art. 10	medical supplies should show that they are safe, of good quality and effective. NRA is National Medicines and Poisons Board	producing country.	of medical devices. No data on import controls	should be recalled, users and patients should be informed.				
WHO[66] (2016)	A medical device "includes an instrument, apparatus, component, part of accessory manufactured or sold for use in the diagnosis, treatment, mitigation or prevention of a disease, disorder or abnormal physical	No information	The Medicines and Allied Substances Act (2013) establishing the NRA: Zambia Medicine Regulatory Authority.	One must obtain marketing authorization from the ZAMRA prior to a medical device's placement on the market, advertisement, manufacture, sell, import, supply, administration, or dealing. Marketing Authorizati	Import controls: A person may not import any allied substance (the definition includes medical devices) without an import permit. Advertising: One may not advertise an allied substance (which includes a medical	No details			No	NRA: Zambia Medicines Regulatory Authority (ZAMRA)

	state or the symptoms of the disease, in human beings or animals[.]" Medicines and Allied Substances Act, Art. 2. Medical devices are included in the definition of "allied substances .		on procedure described in Art. 39 of the Medicines and Allied Substances Act. Clinical investigation controls: No person may conduct a clinical trial involving an allied substance (which includes medical devices) without a clinical trial certificate. The Authority shall keep and maintain a Register of Marketing	device) without a marketing authorization issued by the ZAMRA. Fraudulent, misleading, and deceptive advertisement of allied substances is prohibited. Labelling: Fraudulent, misleading, and deceptive labelling of allied substances is prohibited. Registration of establishment: A person may not manufactu	

				Authorisations issued under the the Medicines and Allied Substances Act	re, distribute, or deal in any allied substance without a license.			
WHO[67] (2016)	Medical device: any device used in the medical field for the purpose of diagnosis, testing, cure, surgery or health protection.	None available	Law 47/2012, Art. 2.7 relating to the regulation food and pharmaceutical products established guidelines for import/export of medicines and other health commodities (includes medical devices). An act of parliament was passed to establish an Inspectorate	Medical devices must meet quality standards and be manufactured in compliance with relevant principles of their manufacture. Any activity related to the manufacture, storing, import or export, sale, packaging, distribution, supply, transport	Import controls: No person shall import and export medical devices unless they are granted a license to do so. Advertising: No person shall label, pack, treat, sell, distribute or advertise any medical device in a manner that is		No	No NRA available yet.

	Authority, Rwanda Inspectorate and Competition Authority (NICA) but it has not yet been implemente d.	medical devices, must be registered. A licence to operate must be granted. No person shall market a pharmace utical product or a medical device on the Rwandan market unless such a product or device is registered. Establishm ents and products are subject to inspection.	false, misleading or is likely to create an erroneous impression regarding its performan ce, design, use, intended use, value or quality.	

Lissel et al[68] (2016)	None provided	Medical device classification systems follow the 4 tier classification system used in the European system. Kenya and SA used the A-D systems while Ghana, Egypt and Ethiopia followed the I-III or I-IV system.	7 countries (Egypt, Ethiopia, SA, Kenya, Ghana, Nigeria and Tanzania) had laws and legal directives that empowered the NRA to regulate medical products in general which may include medical devices. They are also responsible for food and medicines control. The majority of the ABEC countries implemented or harmonized European directives in	Based on EU directives, in order to place a medical device on a market, every device must be compliant to the General Requirements to provide a minimum level of safety for patients and others. To demonstrate compliance with these directives, the relevant standards are applied. Most African		No	NRAs, ABEC.	Most African countries and manufacturers are unable to buy the licenses for these standards needed to show compliance with directives for safety of medical devices. Open Source Medical Devices might be an option to simplify regulatory processes in Africa and save costs while having at least the same safety level. However, even for OSMDs the certification is only valid

			their legislation		countries and manufacturers are unable to buy the licenses for these standards.					for its manufacturer. Every other manufacturer who wants to sell another version of this product on the market must get its own certification.	
Piaggio et al[69] (2020)	None provided	No information		No details	No details	No details	General poor conditions in hospitals were observed around medical devices including high temperatures, humidity, dust and vermin. Electric panels, power transformers, UPS, cables and electric cabins were not installed, maintained or services as expected. There was a lack of standards and a	Findings from these studies show most donated medical devices are not working or not working properly in low resource settings because of poor regulation. HICs define and set standards de facto that cannot be met in most of the lower-	No	A clear need to regulate donations "in a more sensible way" and work towards new standards for medical devices to make them more resilient to harsh environments.	Complete change to more user-driven and contextualized MD design, but also there should be a harmonization of the regulations of medical devices and locations between Europe and Africa. Donations should consider viability and should also be supported by a working local management system. This includes installation and

							failure to meet minimum requirements for use and maintenance of medical devices (structural, organizational and technical)	resource settings.				maintenance support.
Saidi and Douglas[70] (2016)	Same as WHO definition	Most countries used a 4-tier risk based classification system in line with WHO or GHTF recommendations.	All countries studies had some national policy, Act or legislation regarding regulatory oversight of medical products which includes medical devices.	None of the ten African countries discussed have specific regulations or regulatory bodies dedicated to medical devices. All countries performed pre-market regulatory oversight through NRAs charged with oversight of	Various regulatory processes highlighted : pre-import verification of conformity of standards (Kenya); foreign manufacturers providing license evidence and have duly registered agents in-country (Nigeria);specialised expert		The regulations have a strong focus on imports. This is not surprising given heavy reliance on medical devices from developed countries. Few local companies manufacture products for the domestic and export markets. The regulatory approval process is lengthy, not transparent and not as efficient for oversight of these imports as seen in the substandard	Medical device regulations in Africa are designed along the framework of models used in developed countries. For example, the requirements for importation and exportation of medical devices in South Africa, Algeria, Kenya and Ethiopia are similar to the internationally recognised certification/r	No	NRAs	Limited resources and a critical mass of skilled personnel to focus solely on the regulation of medical devices. Under such conditions, the regulatory bodies may fail to cope with registrations of medical devices, which would result in delays and ultimately lack of	Harmonization. Limited regulation a factor hindering access to medical devices in African settings, varying requirements across countries is disincentive for manufacturers and developers

	pharmaceuticals and related medical products, food, and/or cosmetics. Other regulatory organisations included MoH and its Depts and Special Committees in countries. Tanzania and Ethiopia conduct inspection of manufacturing facilities to ensure GMP.	committee reviewing technical documentation for import approval (Egypt); temporary licenses for donated equipment (Sudan); requirements for local representative or vendor authorised to distribute and sell MDs (Algeria, Ethiopia) and issuance of device licenses to manufacturers etc (S/Africa).		MDs seen in the region.	egistration programmes of the European CE Mark, the US FDA and the Australian Hybrid Therapeutic Goods Administration. This is important in that it aligns African countries with a harmonised framework for medical device regulation.		access to medical devices by the public.

Dusabe Gloria[71] (2020)	WHO definition used	Risk based approach to classification was noted in countries with regulatory guidelines	No details. Overall, regulation of medical devices in Africa is limited and the participating African countries were at different maturity levels with respect to existence of medical device regulation, guidelines and actual practice. The WHO Global Model Regulatory Framework for MDs including IVDs medical devices is intended to act as a guide to WHO member	Kenya, Tanzania and Ghana had regulations to guide registration of MDs/IVDs mainly by assessment of technical documents submitted. Uganda and Rwanda had no regulations but relied on international guidelines such as ISO 3485 and USP. All countries recognised the need for revisions of guidelines to increase	Most countries accept approvals based on international guidelines (ISO, US FDA, CE, Health Canada, PMDA Japan) without needing additional regulations in-country. Uganda carries out testing of gloves, condoms and syringes through its Directorate of Lab services.	All countries reported some form of post market vigilance activities of approved or cleared devices happens but this is not often comprehensive. Focus has remained on pre-market approvals, with less emphasis on tracking safety, effectiveness and performance while devices are in use.	Regulatory officers reported receiving basic training in evaluation of MDS- ISO certification, desk review of dossiers, risk based classification, conformity testing etc. Additional training was scheduled based on emerging needs. Training partnerships were formed between South countries as well as Global North and South.	Medical devices are unique and diverse and may require a different regulatory framework than the current medical product approach for oversight in use which is largely focused on pre-market approval. Current regulations may not be clear and subject to gaps in interpretations, or unable to cope with the fast changing pace of technology and development of MDs. Importers have been	No	NRAs	Challenges reported by the participants included inadequate funding and insufficient staffing. In countries without guidelines, delays in establishment of regulatory bodies was experienced with conflict between government sectors on the scope and mandate of control of MDs. More emphasis being placed on pre-market activities and neglect of post market surveillance	Harmonisation efforts: 2 regional blocs were identified working towards harmonisation of regulatory guidelines. These are the EAC and the PAHWP now the African Medical Devices Forum.

		states that plan on setting up regulatory frameworks for MDs.	premarket assessments and post market vigilance. Only 2 countries (Ghana and Tanzania) reported conducting some inspection of manufacturing facilities based on ISO/country guidance.			show to adhere poorly to guidelines.			due to lack of funds to conduct related activities.	
African Union Development Agency [AUDA-NEPAD][72] (2019)	Same as WHO definition	Increased awareness of medical device regulation and established legal frame work and introduction of regulatory framework by more countries	No details	No details	No details	PAHWP's scope of work includes capacity building on; registration and common dossier submissions; quality audit and inspection; clinical performance	No	AUDA, PAWHP, WHO		Improved collaboration, convergence, work sharing and networking amongst members. For example harmonized registration requirements, acceptance of quality management

	was noted in Africa, including adoption of GHTF definition, risk classification, basis for reliance and recognition, requirements for manufactures, declaration of conformity etc.		studies; and post-market surveillance. The PAHWP will also be responsible for conducting training programmes and providing resources to NRAs as part of capacity building.		system auditing reports, shared point for adverse events reports etc. Collaboration with other harmonization initiatives such as AHWP, IMDRF, ASEAN, APEC and any other interested partner should be enhanced.

*Citation of the included source of evidence as it appears in manuscript in superscript.
